# A Bayesian Approach to German Personal and Demonstrative Pronouns

**DOI:** 10.3389/fpsyg.2021.672927

**Published:** 2022-03-03

**Authors:** Clare Patterson, Petra B. Schumacher, Bruno Nicenboim, Johannes Hagen, Andrew Kehler

**Affiliations:** ^1^Department of German Language and Literature I, Linguistics, University of Cologne, Cologne, Germany; ^2^Department of Cognitive Science and Artificial Intelligence, Tilburg School of Humanities and Digital Sciences, Tilburg University, Tilburg, Netherlands; ^3^Department of Linguistics, University of California San Diego, La Jolla, CA, United States

**Keywords:** pronouns, demonstratives, Bayesian model, prominence, reference

## Abstract

When faced with an ambiguous pronoun, an addressee must interpret it by identifying a suitable referent. It has been proposed that the interpretation of pronouns can be captured using Bayes’ Rule: P(referent|pronoun) ∝ P(pronoun|referent)P(referent). This approach has been successful in English and Mandarin Chinese. In this study, we further the cross-linguistic evidence for the Bayesian model by applying it to German personal and demonstrative pronouns, and provide novel quantitative support for the model by assessing model performance in a Bayesian statistical framework that allows implementation of a fully hierarchical structure, providing the most conservative estimates of uncertainty. Data from two story-continuation experiments showed that the Bayesian model overall made more accurate predictions for pronoun interpretation than production and next-mention biases separately. Furthermore, the model accounts for the demonstrative pronoun *dieser* as well as the personal pronoun, despite the demonstrative having different, and more rigid, resolution preferences.

## Introduction

The interpretation of anaphoric pronouns has provided a puzzle for many decades of linguistic research. Third-person anaphoric pronouns such as “she” in (1) are inherently ambiguous in that there are no rigid rules to determine the antecedent. The puzzle for the addressee, then, when faced with a pronoun, is to identify a suitable referent. Despite the ambiguity, this puzzle is solved with ease most of the time: in (1), for example, most people would assume that “she” refers to “the lawyer.”

(1) The lawyer fascinated the judge. She was always so well prepared.

Despite this ease of interpretation, it has proven difficult to accurately describe how pronouns are resolved. It has, however, been possible to identify a range of individual factors which seem to influence resolution; for instance, there is evidence that referents mentioned from subject position are preferred to those mentioned from other positions (Crawley and Stevenson, 1990; Crawley et al., 1990; Gordon et al., 1993; Järvikivi et al., 2005); that referents mentioned first are preferred to those mentioned later (Clark and Sengul, 1979; Gernsbacher and Hargreaves, 1988; Järvikivi et al., 2005); that referents with an agentive thematic role are preferred to those with a patient thematic role (Stevenson et al., 1994; Schumacher et al., 2016); that referents which are topics are preferred to non-topics (e.g., Grosz et al., 1995). These factors also often overlap: in (1), “the lawyer” is both a subject and is mentioned first. The way in which individual factors work together, allowing the addressee to identify the correct referent, however, is still debated.

Describing pronoun resolution as a process that is influenced by a variety of factors allows us to describe certain general tendencies in the language, and can also give insights into the functions of pronouns. But it does not allow us to make precise quantitative predictions about how an addressee will interpret a pronoun in any given context. It is possible to come up with counter-examples for every factor listed above, and influencing factors can be overridden, or at least attenuated, by world knowledge or by coherence relationships between clauses or sentences.

A quite different approach to pronoun interpretation has been taken by Kehler et al. (2008) and Kehler and Rohde (2013). They put forward a simple probabilistic model, the Bayesian model for pronouns, which to a large extent sidesteps the (combination of) individual factors affecting pronoun resolution. Instead, the model makes predictions about how an addressee will interpret a pronoun in a particular linguistic context, by combining the *next-mention bias* with the *production bias*, as described below. Factors influencing the pronoun interpretation do so only indirectly, through their influence on either of the next-mention or production biases (or both).

According to the Bayesian model, addressees reverse-engineer speakers’ intended referents following Bayesian principles:


(2) $$P\,\left(r\,e\,f\,e\,r\,e\,n\,t|p\,r\,o\,n\,o\,u\,n\right)=\frac{P\,\left(p\,r\,o\,n\,o\,u\,n|r\,e\,f\,e\,r\,e\,n\,t\right)\,P\,\left(r\,e\,f\,e\,r\,e\,n\,t\right)}{\sum_{r\,e\,f\,e\,r\,e\,n\,t\in r\,e\,f\,e\,r\,e\,n\,t\,s}P\,\left(p\,r\,o\,n\,o\,u\,n|r\,e\,f\,e\,r\,e\,n\,t\right)\,P\,\left(r\,e\,f\,e\,r\,e\,n\,t\right)}$$

The posterior term P(referent|pronoun) represents the pronoun *interpretation bias*: upon hearing a pronoun (e.g., she), the probability that the addressee will resolve it to a particular referent. The likelihood term P(pronoun|referent) represents the pronoun *production bias*: the probability of the speaker choosing to use a pronoun to refer to an intended referent. Finally, the prior term P(referent) denotes the *next-mention bias*: the probability that a specific referent gets mentioned next by the speaker, regardless of the form of referring expression that they choose. According to this model, therefore, the interpretation and production models are not mirror images of each other, nor is there a simple combination of influencing factors. Instead, pronoun interpretation biases result from an addressee integrating their “top-down” predictions about the content of the ensuing message (particularly, who gets mentioned next) with the “bottom-up” linguistic evidence (particularly, the fact that the speaker opted to use a pronoun).

The performance of the Bayesian model – how well its predictions match actual interpretations – has been compared to the performance of two competing models derived and extended from the existing literature (Ariel, 1990; Gundel et al., 1993; Grosz et al., 1995; Arnold, 1998; inter alia; see Rohde and Kehler, 2014 for discussion). The first we refer to as the Expectancy model, according to which the addressee’s interpretation bias toward a referent is (their estimate of) the probability that the referent is mentioned next in the context. The Expectancy model is inspired by Jennifer Arnold’s claim that a referent’s accessibility is influenced to a considerable extent by the hearer’s estimate of the likelihood that it will be mentioned in the upcoming discourse (Arnold, 1998, 2001). Arnold further developed this insight into the Expectancy Hypothesis (Arnold et al., 2007; Arnold, 2010; Arnold and Tanenhaus, 2011). Arnold (2010) in particular suggests:

*Under the communicative goal of referring, a plausible mechanism for expectancy is as a mechanism for discourse participants to coordinate accessibility. Expectancy describes how easily the comprehender will be able to retrieve the referent. Speakers could thus calculate expectancy as an estimate of accessibility to the listener. (p. 193)*.

This characterization, which is couched in terms of reference production, does not go so far as to claim that pronoun comprehension can be completely equated to the next-mention bias, but it suggests that next-mention bias is a strong influencing factor on the accessibility or activation of a referent, and that this in turn should facilitate pronoun resolution. Our “Expectancy model” instead tests whether the next-mention bias alone guides the predicted interpretation bias, where the next-mention bias P(referent) is normalized by the probabilities of all possible referents that are consistent with the morphological features of the pronoun (e.g., gender, number). This model is mathematically expressed below using the assignment operator to emphasize the fact that this model does not follow normative probability theory.


(3) $$P\,\left(r\,e\,f\,e\,r\,e\,n\,t|p\,r\,o\,n\,o\,u\,n\right)\leftarrow \frac{P\,\left(r\,e\,f\,e\,r\,e\,n\,t\right)}{\sum_{r\,e\,f\,e\,r\,e\,n\,t\in r\,e\,f\,e\,r\,e\,n\,t\,s}P\,\left(r\,e\,f\,e\,r\,e\,n\,t\right)}$$

The second competing model is what we call the Mirror model, according to which the interpretation bias toward a referent is proportional to the likelihood of the referent being pronominalized by the speaker, i.e., the *production bias*. Once again, the assignment operator in (4) reflects the fact that this model does not follow normative probability theory.


(4) $$P\,\left(r\,e\,f\,e\,r\,e\,n\,t|p\,r\,o\,n\,o\,u\,n\right)\leftarrow \frac{P\,\left(p\,r\,o\,n\,o\,u\,n|r\,e\,f\,e\,r\,e\,n\,t\right)}{\sum_{r\,e\,f\,e\,r\,e\,n\,t\in r\,e\,f\,e\,r\,e\,n\,t\,s}P\,\left(p\,r\,o\,n\,o\,u\,n|r\,e\,f\,e\,r\,e\,n\,t\right)}$$

This model captures the idea that addressees will assign interpretations to pronouns by asking what entities the speaker is most likely to refer to using a pronoun instead of a competing referential form. The model is an operationalization of the assumption that pronoun production and pronoun comprehension coordinate on the same notion of entity prominence: that addressees reverse-engineer the speaker’s referential intentions by estimating how likely the speaker is to use a pronoun for a particular referent given its perceived prominence in the discourse context. These estimates therefore rely on a strong correspondence between the form of a referential expression (pronoun, full noun phrase) and the accessibility of its referent. Though this assumption is not often explicitly stated in the psycholinguistics literature, it underlies the treatment of reference production scales being direct representations of mental states, from which assumptions can be made about the salience or accessibility of certain referents (e.g., Ariel, 1990; Gundel et al., 1993). This intuition is cached out by taking the addressee’s estimate of the probability that a speaker will produce a pronoun for a particular referent, normalized by the sum of the probabilities for all compatible referents.

In the current study we assess the performance of the Bayesian model against the two competing models outlined above. Of particular importance is the novel quantitative method used for this assessment. Another novel aspect of this study is the extension of the Bayesian model to German demonstrative pronouns. These pronouns differ from personal pronouns in their resolution biases and therefore provide a good test of the generalizability of the Bayesian model. Furthermore, we go beyond previous assessments of the Bayesian model by testing not only implicit causality verbs (Experiment 2) but also dative-experiencer versus accusative verbs (Experiment 1), in order to explore the influence of grammatical versus thematic roles, which has implications for claims about the strong version of the Bayesian model. Below, we first introduce the strong version of the Bayesian model, and then go on to summarize previous quantitative assessments of the Bayesian model and highlight advantages of the current approach. We then present relevant background on German personal and demonstrative pronouns before stating the study aims.

### Strong Bayesian Model

The primary claim of the Bayesian model is the central prediction underlying equation (2): that comprehenders reverse-engineer the speaker’s referential intentions using Bayesian principles. That is, rather than interpreting pronouns by coordinating with the speaker via a single notion of entity prominence, comprehenders must engage with two types of prominence, one which underlies their estimates of the speaker’s production biases (as captured by the likelihood) and one which underlies their estimates of the next-mention bias (as captured by the prior). It therefore predicts that if independent estimates of the prior, likelihood, and posterior probabilities are obtained, the equation in (2) would approximately hold. We refer to this claim as the *weak* form of the Bayesian model. The model has been successful, for instance, at explaining why in certain contexts, pronoun production biases strongly favor the subject but interpretation biases are more equivocal between potential referents (Source–Goal transfer-of-possession contexts) or even favor the grammatical object (object-biased implicit causality verbs; see Kehler and Rohde, 2013 for discussion).

Kehler et al. (2008) and Kehler and Rohde (2013) also suggested a STRONG version of the Bayesian model, in which the two terms in the numerator of (2) are conditioned by different types of contextual factors. On the one hand, early data had suggested that factors conditioning the next-mention bias P(referent) are primarily semantic and pragmatic in nature (e.g., verb type and coherence relations). On the other hand, the factors that condition the production bias P(pronoun|referent) appear to be grammatical and/or information structural (e.g., based on grammatical role obliqueness or topichood, both of which amount to a preference for sentential subjects). As alluded to above, the resulting prediction, therefore, is that a speaker’s decision about whether or not to pronominalize a referent will be insensitive to a set of semantic and pragmatic contextual factors that the addressee will nonetheless bring to bear via the influence of the prior on interpretation.

Perhaps in the light of the strong, counterintuitive dissociation it posits, it has been the predictions of the strong form of the Bayesian hypothesis that have received the most attention in the literature. Whereas early studies have provided evidence to support it (Rohde, 2008; Fukumura and van Gompel, 2010; Rohde and Kehler, 2014; inter alia), some more recent studies, primarily by Arnold and colleagues, have found limited effects of semantic factors (thematic roles) on production (Rosa and Arnold, 2017; Zerkle and Arnold, 2019; Weatherford and Arnold, 2021; see also Arnold, 2001). These contradictory findings leave us with the looming questions of what the source of the disparities are, and of what type of model can explain the extant data as an ensemble, especially given that the identified effects of semantic factors on production are typically more limited or otherwise inconsistent than theories that rely on a singular notion of entity prominence would predict. It is not the goal of our work to settle this (big) question, but instead to add a new set of facts to the debate by examining the predictions of both the weak and strong models with respect to German personal and demonstrative pronouns.

### Quantitative Assessment of the Bayesian Model

Rohde and Kehler (2014) present the first quantitative evaluation of the Bayesian model against the two competing models (Mirror and Expectancy). They conducted two story-continuation experiments. We describe the method and the materials in detail here, since they are relevant for several aspects of the current study. In a story-continuation experiment, participants are presented with incomplete text passages which they are asked to complete, like those shown in (5) and (6).

(5) a. John scolded Bill. _________
b. John infuriated Bill. _________
c. John chatted with Bill. _________
(6) a. John scolded Bill. He _________
b. John infuriated Bill. He _________
c. John chatted with Bill. He _________

Participants complete the passages, and judges then annotate their continuations. The examples in (5) are the FREE-PROMPT conditions, where just a blank line is presented and participants need to supply the entire sentence. The first referential expression in the participant’s completion is annotated for reference (whether it refers to John or Bill or neither). The form of the referential expression is also annotated, that is, whether the expression itself is a pronoun, a full NP or some other expression. From the annotations of reference in the free-prompt condition, the next-mention bias can be calculated. From the annotation of form combined with reference information, the pronoun production bias for a particular referent (e.g., *John*) can be calculated. From the free-prompt data, then, predictions for all three models described in the previous section can be derived. The PRONOUN-PROMPT conditions are shown in (6). Here, instead of a blank line, a pronoun is presented in first position and the participant supplies the rest of the sentence. In these conditions the reference for the pronoun is annotated, yielding the actual interpretation bias for the pronoun. As such, the models’ predicted interpretation bias as derived from the free-prompt data can be compared against actual interpretation bias measured from the pronoun-prompt data.

Using this method, Rohde and Kehler (2014; see also Rohde, 2008) tested whether the next-mention bias (i.e., the prior) and production bias (i.e., the likelihood) were sensitive to semantic biases arising from implicit causality (IC), that is, they tested the strong form of the Bayesian model. For example, a subject-biased IC verb such as *infuriate* as in (5b/6b) implies that the subject *John* is the cause of the infuriation event, while an object-biased IC verb such as *scold* as in (5a/6a) implies that the object *Bill* is the cause of the scolding event. In Rohde and Kehler’s experiment, the IC verbs were compared to neutral (non-IC) verbs such as *chat with* as in (5c/6c). As predicted by the strong Bayesian hypothesis, the verb type affected both the next mention biases in the free condition (5) and the pronoun interpretation biases in the pronoun-prompt condition (6), with subject mentions in both prompt conditions being most frequent for subject-biased IC contexts, least frequent for object-biased IC contexts, and in between for non-IC controls. However, the difference in subject next-mention rate was not coupled with a difference in pronominalization rates for subject next-mentions in the free-prompt conditions. Instead, only the grammatical role of the referent’s previous mention mattered: participants pronominalized references to the previous subject far more often than ones to the previous non-subject. To put a fine point on this, participants were no more likely to pronominalize a mention of the previous object in an object-biased IC context like (5a) than in a subject-biased IC one like (5b), and similarly no more likely to pronominalize a mention of the previous subject in a subject-biased context (5b) than in an object-biased one (5a).

For both experiments, predictions per participant and per item for the Bayesian, Mirror and Expectancy models were generated as described above. These predictions were correlated against per participant and per item actual observations from the pronoun-prompt condition and the correlations were evaluated using R^2^. While the predictions of all the models were significantly correlated with the observed data, the Bayesian model consistently produced the strongest correlations.

Zhan et al. (2020) were able to improve on the assessment of model performance presented in Rohde and Kehler (2014) by combining R^2^ with MSE and ACE metrics. MSE and ACE weigh different aspects of model performance; while ACE reflects discrepancies between predicted and observed behavior at extreme values, MSE reflects discrepancies throughout the range of values. A downside of their approach, however, is that the predictions are based on point estimates and do not take into account the uncertainty in the data. The measures of discrepancy ignore the inherent noisiness of the data that were used to make model predictions and might give overoptimistic estimates as a result^1^. In the analysis presented in this paper, we used Bayesian methods that propagate the uncertainty in the data to the predictions. Rather than point-values, we predict distributions of possible values. The width of the prediction distribution depends on the uncertainty (or variability) present in the data. This approach thus makes a new contribution to the assessment of pronoun interpretation models.

### Cross-Linguistic Support for the Bayesian Model

The Bayesian model for pronouns has, for the most part, been developed and tested on English (Kehler et al., 2008; Kehler and Rohde, 2013; Rohde and Kehler, 2014), while cross-linguistic support is only now starting to emerge (Bader and Portele, 2019; Zhan et al., 2020). While there is nothing about the model’s mechanics that make it specific to one language, it remains to be seen whether claims associated with the model are applicable in other languages. Zhan et al. (2020) tested subject-biased and object-biased IC verbs using the same story continuation task as Rohde and Kehler (2014). They replicated the effect of verb type on the next-mention bias and the effect of grammatical role (and not verb type) on the pronoun production biases, in line with the strong Bayesian model. Furthermore, their results also indicated that grammatical role rather than topichood affects the pronoun production biases, in contrast to Rohde and Kehler (2014).

It is also important to test the model in different pronoun systems. This was not a feature of the Zhan et al., study; while Mandarin Chinese has both null and overt pronouns, they appear to have largely overlapping resolution preferences. It is possible, for example, that the Bayesian model is better suited to making predictions for pronouns whose interpretation is quite flexible. It remains to be seen whether a pronoun with more rigid preferences can be accounted for equally well. We address this question by testing the Bayesian model on the German personal pronoun *er* and the demonstrative pronoun *dieser*. Below, we briefly outline the relevant properties of these pronouns and also consider the findings of Bader and Portele (2019), who incorporated aspects of the Bayesian model into their study on the German demonstrative *der*. We then set out the goals of this paper before reporting our experiments.

### German Personal and Demonstrative Pronouns

German personal pronouns, for example *er* (‘‘he’’), are quite similar to English personal pronouns, but unlike English they can be used to refer to both animate and inanimate entities. In addition, German has a rich set of demonstratives that can be used pronominally, for example *der*, *dieser*, *jener*, *derjenige*. When functioning as pronominals (as opposed to adnominals, e.g., *dieser Mann* ‘‘this man’’), these demonstratives can refer to animate or inanimate entities just like personal pronouns^2^.

When referring to animate entities, German personal and demonstrative pronouns tend to differ regarding both interpretative preferences and their influence on maintenance and shift of the sentence topic (see Schumacher et al., 2015, 2016; Portele and Bader, 2016; Fuchs and Schumacher, 2020). Most previous research on interpretive preferences has looked at *der* compared to *er*, while *dieser* has received far less attention. It has been claimed that the personal pronoun *er* has a bias toward subject referents (Bosch et al., 2003, 2007; Bouma and Hopp, 2006, 2007) while *der* has been described as object-biased (Kaiser, 2011) and as having an anti-topic bias (Bosch and Umbach, 2007; Wilson, 2009; Hinterwimmer, 2015; Bosch and Hinterwimmer, 2016). Nonetheless, the personal pronoun appears to be quite flexible; the demonstrative *der*, on the other hand, seems to be less flexible (Kaiser, 2011; Schumacher et al., 2015, 2016, 2017; Bader and Portele, 2019). Patil et al. (2020) examined the demonstrative *dieser* and found an anti-subject preference; they proposed that *dieser* is the formal counterpart of *der*. The contrast in flexibility of interpretation between personal and demonstrative pronouns allows us to explore whether the Bayesian model, in which the prior can move biases around, can also be applied to a more “rigid” pronoun.

Bader and Portele (2019), in a series of story-continuation experiments, found that subjecthood had the strongest impact on interpretation of *er*, while interpretation of *der* was influenced to some extent by subjecthood, topichood and linear order. They also used their data to assess the predictions of the Bayesian model. In a separate experiment participants were presented with the items from the first two experiments with just the free-prompt for story completion^3^. However, the experimental materials were more complex than in previous story-continuation experiments (e.g., Rohde and Kehler, 2014; Zhan et al., 2020), because items started with a context sentence in which a (feminine) referent was introduced before the critical sentence containing the two (masculine) entities which were potential referents for the pronouns tested. While the entity in the context sentence was not a potential referent for the pronoun in the pronoun-prompt conditions, it was nevertheless referred to in 49% of completions in the free-prompt condition (i.e., when the prompt contained no pronoun). This introduced an imbalance in the available observations. In fact, P(referent) was calculated using all observations (including references to the entity in the context sentence and to both entities) while the sum of production probabilities used in the Bayesian calculation was only from NP1 and NP2. We suspect this may have led to an imbalance in the calculation of predictions for the Bayesian model. They report a high R^2^ value (0.95) for the correlation between predicted and observed values^4^, but we think that this result should be interpreted with caution. Performance of competing models (Expectancy and Mirror) were not reported.

One further aspect of *der* (and *dieser*) demonstratives that should be highlighted is the potential role of agentivity. A series of studies by Schumacher et al. (2015, 2016, 2017) and Fuchs and Schumacher (2020) has shown that agentivity is an important factor for personal and demonstrative pronouns in German. This has been shown by contrasting verbs in which thematic roles and grammatical roles align with verbs in which they are not aligned. For example, in accusative verbs such as *ärgern* ‘‘annoy,’’ agentivity and grammatical role are aligned because the subject of the verb has the proto-agent role and the object the proto-patient role^5^. In contrast, in dative-experiencer verbs such as *imponieren* “impress,” agentivity and grammatical role are not aligned because the object has the proto-agent role and the subject has the proto-patient role (note also that in canonical order the object, not the subject, is in initial position). In other words, the grammatical role hierarchy (subject > object) and thematic role hierarchy (proto-agent > proto-patient) are aligned in the accusative verbs and not aligned in the dative-experiencer verbs. Pronoun interpretation in these experiments was affected to a greater degree by agentivity than by grammatical role, with personal pronouns tending to refer to the proto-agent and demonstratives to the proto-patient. Given this finding, we decided to exploit this verb-type contrast to explore the relative influence of agentivity and subjecthood on production biases in German. While the strong form of the Bayesian model specifies that subjecthood and/or topichood influences production likelihoods (Rohde and Kehler, 2014; Zhan et al., 2020), it is possible that in German agentivity also has an influence, in the light of Schumacher and colleagues’ findings about the influence of agentivity on interpretation^6^.

For the current study, we chose to focus on the demonstrative *dieser* as opposed to *der* for two reasons. First, *dieser* is better suited to a written experiment than *der*, which is perceived by some speakers to be slightly pejorative and is more appropriate in spoken, possibly less formal, contexts^7^. This is supported by Bader and Portele’s (2019) experiments in which *dieser* was elicited far more frequently than *der* in the free-prompt conditions. Second, little is known about general interpretive preferences for *dieser* since most previous studies have looked at *der*; descriptions of *dieser* in German grammars are brief and empirically inadequate (but see Fuchs and Schumacher, 2020 for a recent comparison of *der* and *dieser*). It would therefore be useful to expand our understanding of how *dieser* differs from the personal pronoun in German.

## Current Study

The purpose of the current study is to assess the performance of the Bayesian model on German personal and demonstrative pronouns, in order to address the following main questions:

• Which model for pronouns (Bayesian, Expectancy or Mirror) best accounts for the interpretation of German personal and demonstrative pronouns?
• Is the resolution of demonstratives as rigid as some previous studies suggest, or is the interpretation influenced by the next-mention bias, as the Bayesian model would predict?
• Is there evidence for the strong form of the Bayesian model?

In the following, we present two text completion experiments that address these questions. We use the free-prompt data to generate predictions for the Bayesian, Mirror and Expectancy models and compare the predictions to the observations from the pronoun-prompt conditions. Model predictions are generated in a Bayesian statistical framework with a fully hierarchical structure and weakly informative priors. The hierarchical structure allows us to accommodate, for example, participant and item effects directly in our model predictions without having to average over them. In contrast to previous evaluations of model performance, the Bayesian statistical approach allows us to estimate the parameters of a distribution of predicted observations, allowing us to make more stable inferences about model performance.

## Experiment 1

Experiment 1 was a text continuation task testing the next-mention, production and interpretation biases associated with the German personal pronoun *er* and the demonstrative pronoun *dieser*, in contexts with accusative verbs and dative-experiencer verbs. In addition to addressing the main questions set out above, our motivation for the verb-type contrast was to explore the relative contribution of agentivity and subjecthood to the production likelihoods. A strong influence of agentivity would be seen in higher pronoun production likelihoods for proto-agents than for proto-patients for personal pronouns, and the opposite pattern for demonstratives. Proto-agents are the first NP (henceforth NP1) in both verb types. A strong influence of subjecthood, in contrast, would result in higher personal production likelihoods for the grammatical subject, which is NP1 for accusative verbs and NP2 for the dative verbs. For the demonstrative, a grammatical role influence would result in higher production likelihoods for NP2 in accusative verbs and NP1 in dative verbs.

### Participants

Fifty nine participants from the University of Cologne took part in Experiment 1. Nine participants were excluded because they did not complete the experiment (less than 75% of items completed); one participant was excluded for not following the task instructions and one participant was excluded for lack of German knowledge. Data from the remaining 48 participants (39 female, 7 male, 2 gender not indicated) were used in the analysis. All 48 participants indicated that they were German native speakers; 7 participants were bilingual. No participants reported language-related disorders.

### Materials

Seventy two critical items were constructed, each in three prompt conditions: *er*, *dieser* or a free-prompt (blank line); see (7) and (8). A full list of items and fillers is available on OSF^8^. Critical items consisted of a context sentence followed by the prompt. The context sentences consisted of a main clause with two masculine animate arguments, starting with an adjunct (e.g., *vorletzte Nacht* ‘‘the night before last’’). The main verb in the context sentences was either an accusative or a dative-experiencer verb (henceforth ‘‘dative’’), always in the perfect tense (comprising a form of *sein* ‘‘to be’’ or *haben* ‘‘to have’’ plus a participle). Context sentences were always presented in canonical argument order (proto-agent before proto-patient, i.e., nominative--accusative for the accusative verbs and dative--nominative for the dative verbs). The 36 accusative items contained 36 different verbs, but the 36 dative items were limited to just four verbs which were re-used^9^.

(7) Accusative items:

(a) *Er prompt:* Nach dem Fußballspiel hat der Franzose den Italiener gesehen. Er _________
(b) *Dieser prompt:* Nach dem Fußballspiel hat der Franzose den Italiener gesehen. Dieser _________
(c) *Free-prompt:* Nach dem Fußballspiel hat der Franzose den Italiener gesehen. _________

“After the football game the Frenchman *(nom.masc.)* saw the Italian (*acc.masc.*). *He/DEM/*…”

(8) Dative items:

(a) *Er prompt:* Gestern ist dem Feuerwehrmann der Polizist aufgefallen. Er _________
(b) *Dieser prompt:* Gestern ist dem Feuerwehrmann der Polizist aufgefallen. Dieser _________
(c) *Free-prompt:* Gestern ist dem Feuerwehrmann der Polizist aufgefallen. _________

“Yesterday the firefighter *(dat.masc.)* noticed the police officer (*nom.masc.*). *He/DEM/*…”

Role names (e.g., *Polizist* “police officer”) with masculine gender were used for both entities introduced in the context sentence in all but two items, in which animals (also masculine) were used. Hierarchical relationships between the two roles (such as teacher–pupil) were avoided to prevent a prominence confound. The pronouns in the pronoun-prompt conditions always matched in gender with the entities in the context sentence so that both were potential referents for the pronoun. Note that feminine pronouns/referents were not tested, because the feminine personal pronoun *sie* in German is ambiguous in terms of case and number.

The 72 item-sets were mixed with 30 “true” fillers (25% gender-ambiguous, 50% gender-disambiguated and 25% items with one referent only) and 6 “catch” fillers (included to ensure that participants were paying attention to the task), and distributed over three lists in a Latin-square design. Ten of the fillers contained target sentences that began with a temporal adverbial (five items) or connector (five items), and ten contained target sentences with a connector followed by an auxiliary and a pronoun. Another ten filler items comprised personal or demonstrative pronoun-prompts in the style of the critical items. Two of the demonstrative filler items, which were presented among the first ten items, included an auxiliary or adverb after the pronoun-prompt (*Dieser ist* ____, “He.dem is”; *Trotzdem haben diese dann* ____, “Nevertheless they.dem have then”) which forces a pronominal reading of the demonstrative. The aim was to prime the participants to produce a pronominal, as opposed to an adnominal, use of the demonstrative (Bader and Portele, 2019, reported very low uses of the demonstrative pronoun in the free-prompt condition, and Kaiser, 2011, reports 75.6% completions with an adnominal use of the demonstrative).

### Procedure

The lists were presented to participants in a seminar setting as a paper questionnaire comprising 108 items. The first page contained study information and a consent form. Participants then answered a short series of biographical questions before starting the experimental task. Participants were instructed to complete every short story by supplying the second sentence, without making changes to the text presented. They were additionally instructed that the most obvious completion should be written and not the most creative or humorous one, and that completions should be kept short and precise.

### Data Coding

The data was coded by two native speakers of German; one Linguistics Masters student and one technical assistant. Coder 1 identified missing and ungrammatical continuations which were excluded from the analysis. Both annotators made independent judgments about the intended referent of the first referential expression (in the pronoun-prompt conditions, the first referential expression was always the pronoun given in the prompt, i.e., *er* or *dieser*). The referent for the first referential expression was coded in five categories: NP1, NP2, both, neither, ambiguous. The two annotators agreed in 77% of observations, with a Cohen’s (unweighted) Kappa of 0.669 (*z* = 70.8, *p* ≤ 0.001). Observations where the annotators disagreed were resolved through discussion to produce a final data set for analysis. The first referential expression in the free-prompt data was also categorized. Data from 48 participants for 24 items (12 accusative, 12 dative) per prompt condition resulted in a total of 3456 observations; 1152 in each of the *er*-, *dieser*- and free-prompt conditions. The distribution of reference and the response categories for the first referential expression are given in Supplementary Material. For the following analyses, the dataset was reduced by dropping cases that were missing, ungrammatical, and references that were ambiguous, plural, complex (referring to a whole event or proposition), or where no referential expression occurred or cases where the first expression was an impersonal pronoun, leaving a total of 2390 observations for the analysis (679 free-prompt, 858 *er*-prompt and 853 *dieser*-prompt).

### Data Analysis

For the data analysis and modeling, we use a Bayesian data analysis approach implemented in the probabilistic programming language *Stan* (Stan Development Team, 2020) in *R* (R Core Team, 2020)^10^. An important motivation for using the Bayesian approach is that it allows us to implement a fully hierarchical structure to any type of model (e.g., the so-called “maximal random effect structure”); a hierarchical structure provides the most conservative estimates of uncertainty (Schielzeth and Forstmeier, 2008). In all our models, we use regularizing priors, which we detail below. These priors are minimally informative and have the objective of yielding stable inferences (Gelman et al., 2008; Chung et al., 2015). Nicenboim and Vasishth (2016) and Vasishth et al. (2018) discuss the Bayesian approach in detail in the context of psycholinguistic and phonetic sciences research. We fit the models with four chains and 4000 iterations each, of which 1000 iterations were the burn-in or warm-up phase. In order to assess convergence, we verify that there are no divergent transitions, that all the $\hat{R}$ (the between- to within-chain variances) are close to one, that the number of effective sample size are at least 10% of the number of post-warmup samples, and we visually inspect the chains.

As we detail below, the models fit the produced referents (NP1 or NP2, discarding the ambiguous or other referents) with a Bernoulli likelihood, where its parameter θ is fitted in log-odds space, and/or the produced pronoun type (personal or demonstrative pronoun, or other expressions) with a categorical likelihood. The probability of a personal pronoun and “other” with respect to the reference category, demonstrative pronoun, is also fitted in log-odds space (that is, the categorical likelihood is composed of two equations that contrast the odds of producing a personal pronoun or other expression instead of the reference category, demonstrative pronouns). For more details about categorical or multinomial logistic regression see Koster and McElreath (2017). For both the Bernoulli and the categorical regressions, we assume a hierarchical structure composed of an intercept denoted by α, a number of slopes denoted by β, and a number of by-participant and by-item adjustments to the intercept and slope, *u* and *w*, respectively. All these parameters have the following weakly regularizing priors:

• The intercepts of the Bernoulli (α) have priors in probability space: *logit^–1^(α)* ∼ *Beta*(1,1).
• The intercept of the equations in the categorical regression (α) have *Normal*(0, 2) priors.
• All the slopes (β) have as a prior *Normal*(0, 2).
• All the variance components of the by-group adjustments (or random effects) are *Normal*_+_(0, 2).
• The correlations between by-participants and by-items adjustments have each *lkj*(2) as a prior.

For each model we report the mean estimates and 95% quantile-based Bayesian credible intervals of the main parameters. A 95% Bayesian credible interval has the following interpretation: it is an interval containing the true value with 95% probability given the data and the model (see, for example Jaynes and Kempthorne, 1976; Morey et al., 2016). We evaluate the fit of models graphically with holdout predictive check, and numerically using holdout validation (Vehtari and Ojanen, 2012). Crucially, we evaluate the performance of the different models with respect to their predictive accuracy on new data that is *never used to estimate the parameters*. An advantage of model comparison based on hierarchical Bayesian models is that the uncertainty of the models’ parameters is propagated to the predictions that they make: This means that instead of point predictions, the models generate a distribution of predictions. For holdout validation, we compare the models based on their pointwise log predictive density^11^.

### Results

**FIGURE 1:**
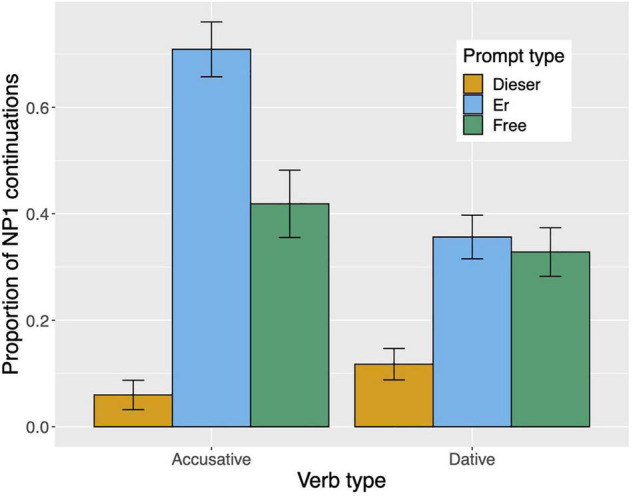
Proportion of NP1 continuations per prompt condition (*er, dieser, and free*) and verb type in Experiment 1. The NP1 continuations represent the next-mention bias for NP1. Error bars represent 95% confidence intervals based on normalized data following Morey (2008) and Chang (2013).

**FIGURE 2:**
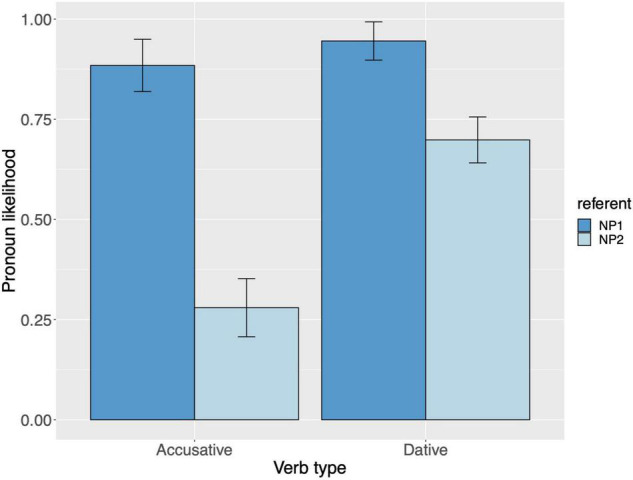
Personal pronoun: probability of using a personal pronoun to refer to NP1 and NP2, per verb type, for Experiment 1. These bars represent the pronoun production likelihoods (and are therefore based on the free-prompt data). Error bars are by-participant and represent 95% confidence intervals based on normalized data following Morey (2008) and Chang (2013).

**FIGURE 3:**
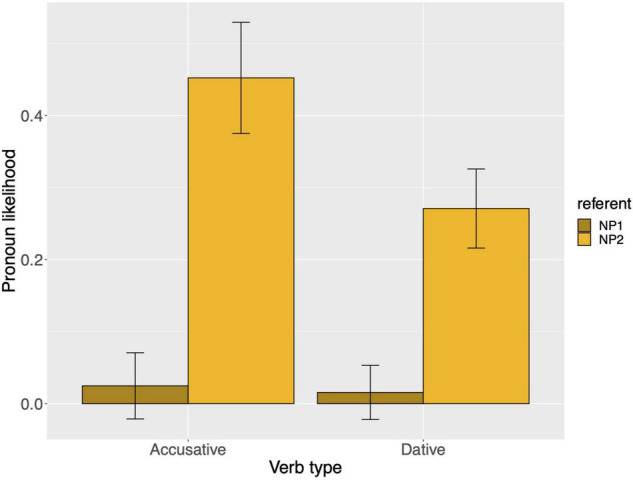
Demonstrative pronoun: probability of using a demonstrative pronoun to refer to NP1 and NP2, per verb type, for Experiment 1. These bars represent the pronoun production likelihoods (and are therefore based on the free-prompt data). Error bars are by-participant and represent 95% confidence intervals based on normalized data following Morey (2008) and Chang (2013).

Raw proportions for the next-mention bias are shown in Figure 1. Figure 2 shows the personal pronoun production likelihoods, and Figure 3 the demonstrative pronoun production likelihoods^12^. When calculating likelihoods for the personal pronoun, both subject and non-subject personal pronouns were included. For the demonstrative pronoun, both subject and non-subject demonstrative pronoun *dieser*, and subject and non-subject demonstrative pronoun *der*, were included.

#### Modeling

##### Expectancy Model

The Expectancy model predicts that the probability of referring to NP1 in the pronoun-prompt data is determined by the prior probability of NP1 (*P*(*referent = NP1*)). This prior can be estimated from the free-prompt data. The Expectancy model was built in the following way and its parameters were estimated using only the free-prompt data:


$$\eta_{i}=\alpha_{N\,P\,1}+u_{N\,P\,1}\,\left[s\,u\,b\,j\,\_\,f\,r\,e\,e\,\left[i\right]\right]+w_{N\,P\,1}\,\left[i\,t\,e\,m\,\_\,f\,r\,e\,e\,\left[i\right]\right]+v\,t\,y\,p\,e\,\left[i\right]\cdot \left(\beta_{v\,t\,y\,p\,e}+u_{v\,t\,y\,p\,e}\,\left[s\,u\,b\,j\,\_\,f\,r\,e\,e\,\left[i\right]\right]\right)$$

(9) $$P\left(NP1|\ldots \right)=P(referent=NP1|item\_free\left[i\right],subj\_free\left[i\right],vtype_{i})=logit{}^{-1}(\eta_{i})N\,P\,1_{i}\,\sim \,B\,e\,r\,n\,o\,u\,l\,l\,i\,\left(P\,\left(N\,P\,1|\ldots \right)\right)$$

where *NP1* is 1 if the referent is NP1 and 0 if the referent is NP2, *i* indicates the observation of the free-prompt data, *vtype* is a vector that maps between observations and the corresponding verb type (accusative coded with 1 or dative coded with −1), *subj_free* and *item_free* are vectors that indicate the mapping between observations, and subjects and items, respectively, and *u* and *w* are the by-subject and by-items adjustments (or “random effects”). The three dots (…) symbolize all the information that the model is taking into account to estimate the probability of producing NP1 as a referent: the characteristics of the stimuli (i.e., intercept, beta, and by-item adjustments) and of the subject performing the free-prompt task (i.e., by-subject adjustments).

The parameters estimated with the free-prompt data were used to generate predictions for the pronoun-prompt data in the following way:


$$\eta_{n}=\alpha_{N\,P\,1}+u_{N\,P\,1}\,\left[s\,u\,b\,j\,\_\,p\,r\,o\,n\,\left[n\right]\right]+w_{N\,P\,1}\,\left[i\,t\,e\,m\,\_\,p\,r\,o\,n\,\left[n\right]\right]+v\,t\,y\,p\,e\,\left[n\right]\cdot \left(\beta_{v\,t\,y\,p\,e}+u_{v\,t\,y\,p\,e}\,\left[s\,u\,b\,j\,\_\,p\,r\,o\,n\,\left[n\right]\right]\right)$$

(10) $$P\left(NP1|\ldots \right)=P(referent=NP1|item\_pron\left[n\right],subj\_pron\left[n\right],vtype_{n})=logit{}^{-1}(\eta_{n})p\,r\,e\,d_{N\,P\,1_{n}}\,\sim \,B\,e\,r\,n\,o\,u\,l\,l\,i\,\left(P\,\left(N\,P\,1|\ldots \right)\right)$$

where *n* indicates the observation of the pronoun-prompt data, *subj_pron* and *item_pron* are vectors that indicate the mapping between observations for subjects and items, respectively, and *u* and *w* are the by-subject and by-items adjustments. As before, the three dots (…) symbolize all the information that the model is taking into account to generate the predictions: the characteristics of the stimuli (i.e., intercept, beta, and by-item adjustments) and of the subject performing the pronoun-prompt task (i.e., by-subject adjustments).

##### Mirror Model

The Mirror model predicts that the probability of referring to *NP1* for pronoun-prompt data is determined by the *likelihood* of NP1 (*P(pronoun|referent = NP1)*) normalized to be a probability distribution by dividing the likelihood by the marginal probability distribution of the pronouns. This normalized *likelihood* can be estimated from the free-prompt data. The Mirror model was built in the following way:


$$\text{log}\,\left(\frac{\theta_{P\,P_{i}}}{\theta_{D\,P_{i}}}\right)=\alpha_{P\,P}+u_{P\,P}\,\left[s\,u\,b\,j\,\_\,f\,r\,e\,e\,\left[i\right]\right]+w_{P\,P}\,\left[i\,t\,e\,m\,\_\,f\,r\,e\,e\,\left[i\right]\right]$$

$$+v\,t\,y\,p\,e\,\left[i\right]\cdot \left(\beta_{P\,P,v\,t\,y\,p\,e}+u_{P\,P,v\,t\,y\,p\,e}\,\left[s\,u\,b\,j\,\_\,f\,r\,e\,e\,\left[i\right]\right]\right)$$

$$+ref\_free_{i}\cdot (\beta_{P\,P,r\,e\,f}+u_{P\,P,r\,e\,f}\left[subj\_free\left[i\right]\right]$$

$$+w_{P\,P,r\,e\,f}\left[item\_free\left[i\right]\right])+vtype[i]\cdot ref\_free{}_{i}\cdot (\beta_{P\,P,i\,n\,t}$$

$$+u_{P\,P,i\,n\,t}\left[subj\_free\left[i\right]\right]+w_{P\,P,i\,n\,t}\left[item\_free\left[i\right]\right])$$

$$l\,o\,g\,\left(\frac{\theta_{D\,P_{i}}}{\theta_{D\,P_{i}}}\right)=0$$

$$l\,o\,g\,\left(\frac{\theta_{o\,t\,h\,e\,r_{i}}}{\theta_{D\,P_{i}}}\right)=\alpha_{o\,t\,h\,e\,r}+u_{o\,t\,h\,e\,r}\,\left[s\,u\,b\,j\,\_\,f\,r\,e\,e\,\left[i\right]\right]$$

$$+w_{o\,t\,h\,e\,r}\left[item\_free\left[i\right]\right]+vtype\left[i\right]\cdot (\beta_{o\,t\,h\,e\,r,v\,t\,y\,p\,e}$$

$$+u_{o\,t\,h\,e\,r,v\,t\,y\,p\,e}\left[subj\_free\left[i\right]\right])+ref\_free{}_{i}\cdot (\beta_{o\,t\,h\,e\,r,r\,e\,f}$$

$$+u_{o\,t\,h\,e\,r,r\,e\,f}\left[subj\_free\left[i\right]\right]+w_{o\,t\,h\,e\,r,r\,e\,f}\left[item\_free\left[i\right]\right])$$

$$+vtype\left[i\right]\cdot ref\_free_{i}\cdot (\beta_{o\,t\,h\,e\,r,i\,n\,t}$$

$$+u_{o\,t\,h\,e\,r,i\,n\,t}\left[subj\_free\left[i\right]\right]+w_{o\,t\,h\,e\,r,i\,n\,t}\left[item\_free\left[i\right]\right])$$

(11) $$p\,r\,o\,n_{i}∼C\,a\,t\,e\,g\,o\,r\,i\,c\,a\,l\,\left(\theta_{P\,P_{i}},\theta_{D\,P_{i}},\theta_{o\,t\,h\,e\,r_{i}}\right)$$

where *pron* is 1 if the free completion includes a personal pronoun, 2 if it includes a demonstrative pronoun, and 3 otherwise; *i* indicates the observation of the free-prompt data, *vtype* is vector that maps between observations and the corresponding verb type (accusative coded as 1 or dative coded as −1), *ref_free* indicates whether the referent of the completion is NP1 (coded with 1) or NP2 (coded with −1), and, just as for the Expectancy model, *subj_free* and *item_free* are vectors that indicate the mapping between the observations and subjects or items, respectively, and *u* and *w* are the by-subject and by-items adjustments. The parameters estimated with the free-prompt data were used to generate predictions for each observation *n* of the pronoun-prompt data as described below.

First, the likelihood of each referent is calculated. To simplify the equations, we define:


P(PP|NP1,…)=P(pronoun=PP|referent=NP1,subj_pron[n],item_pron[n],vtype[n])

P(PP|NP2,…)=P(pronoun=PP|referent=NP2,subj_pron[n],item_pron[n],vtype[n])

P(DP|NP1,…)=P(pronoun=DP|referent=NP1,subj_pron[n],item_pron[n],vtype[n])

P(DP|NP2,…)=P(pronoun=DP|referent=NP2,subj_pron[n],item_pron[n],vtype[n])

P(other|NP1,…)=P(pronoun=other|referent=NP1,subj_pron[n],item_pron[n],vtype[n])

P(other|NP2,…)=P(pronoun=other|referent=NP2,subj_pron[n],item_pron[n],vtype[n])

P(NP1|PP,…)=P(referent=NP1|pronoun=PP,subj_pron[n],item_pron[n],vtype[n])

(12) P(NP1|DP,…)=P(referent=NP1|pronoun=DP,subj_pron[n],item_pron[n],vtype[n])

<P(PP|NP1,…),P(DP|NP1,…),P(other|NP1,…)>

$$=softmax(\,\alpha_{P\,P}+u_{P\,P}\,\left[s\,u\,b\,j\,\_\,p\,r\,o\,n\,\left[n\right]\right]+w_{P\,P}\,\left[i\,t\,e\,m\,\_\,p\,r\,o\,n\,\left[n\right]\right]\;+v\,t\,y\,p\,e\,\left[n\right]\cdot \left(\beta_{P\,P,v\,t\,y\,p\,e}+u_{P\,P,v\,t\,y\,p\,e}\,\left[s\,u\,b\,j\,\_\,p\,r\,o\,n\,\left[n\right]\right]\right)$$

$$+\left(\beta_{P\,P,r\,e\,f}+u_{P\,P,r\,e\,f}\,\left[s\,u\,b\,j\,\_\,p\,r\,o\,n\,\left[n\right]\right]+w_{P\,P,r\,e\,f}\,\left[i\,t\,e\,m\,\_\,p\,r\,o\,n\,\left[n\right]\right]\right)+vtype\left[n\right]\cdot (\beta_{P\,P,i\,n\,t}+u_{P\,P,i\,n\,t}\left[subj\_pron\left[n\right]\right]$$

$$+w_{P\,P,i\,n\,t}\left[item\_pron\left[n\right]\right]),\,0,$$

$$\,\alpha_{o\,t\,h\,e\,r}+u_{o\,t\,h\,e\,r}\,\left[s\,u\,b\,j\,\_\,p\,r\,o\,n\,\left[n\right]\right]+w_{o\,t\,h\,e\,r}\,\left[i\,t\,e\,m\,\_\,p\,r\,o\,n\,\left[n\right]\right]+v\,t\,y\,p\,e\,\left[n\right]\cdot \left(\beta_{o\,t\,h\,e\,r,v\,t\,y\,p\,e}+u_{o\,t\,h\,e\,r,v\,t\,y\,p\,e}\,\left[s\,u\,b\,j\,\_\,p\,r\,o\,n\,\left[n\right]\right]\right)$$

$$+(\beta_{o\,t\,h\,e\,r,r\,e\,f}+u_{o\,t\,h\,e\,r,r\,e\,f}\left[subj\_pron\left[n\right]\right]+w_{o\,t\,h\,e\,r,r\,e\,f}\left[item\_pron\left[n\right]\right])+vtype\left[n\right]\cdot (\beta_{o\,t\,h\,e\,r,i\,n\,t}+u_{o\,t\,h\,e\,r,i\,n\,t}\left[subj\_pron\left[n\right]\right]$$

$$+w_{o\,t\,h\,e\,r,i\,n\,t}\,\left[i\,t\,e\,m\,\_\,p\,r\,o\,n\,\left[n\right]\right])<P\left(PP|NP2,\ldots \right),P\left(DP|NP2,\ldots \right),P\left(other|NP2,\ldots \right)>$$

$$=softmax(\,\alpha_{P\,P}+u_{P\,P}\,\left[s\,u\,b\,j\,\_\,p\,r\,o\,n\,\left[n\right]\right]+w_{P\,P}\,\left[i\,t\,e\,m\,\_\,p\,r\,o\,n\,\left[n\right]\right]+v\,t\,y\,p\,e\,\left[n\right]\cdot \left(\beta_{P\,P,v\,t\,y\,p\,e}+u_{P\,P,v\,t\,y\,p\,e}\,\left[s\,u\,b\,j\,\_\,p\,r\,o\,n\,\left[n\right]\right]\right)$$

$$+\left(-1\right)\cdot (\beta_{P\,P,r\,e\,f}+u_{P\,P,r\,e\,f}\left[subj\_pron\left[n\right]\right]+w_{P\,P,r\,e\,f}\left[item\_pron\left[n\right]\right])$$

$$+vtype\left[n\right]\cdot \left(-1\right)\cdot (\beta_{P\,P,i\,n\,t}+u_{P\,P,i\,n\,t}\left[subj\_pron\left[n\right]\right]+w_{P\,P,i\,n\,t}\left[item\_pron\left[n\right]\right]),\,0,$$

$$\,\alpha_{o\,t\,h\,e\,r}+u_{o\,t\,h\,e\,r}\,\left[s\,u\,b\,j\,\_\,p\,r\,o\,n\,\left[n\right]\right]+w_{o\,t\,h\,e\,r}\,\left[i\,t\,e\,m\,\_\,p\,r\,o\,n\,\left[n\right]\right]+v\,t\,y\,p\,e\,\left[n\right]\cdot \left(\beta_{o\,t\,h\,e\,r,v\,t\,y\,p\,e}+u_{o\,t\,h\,e\,r,v\,t\,y\,p\,e}\,\left[s\,u\,b\,j\,\_\,p\,r\,o\,n\,\left[n\right]\right]\right)$$

$$+\left(-1\right)\cdot (\beta_{o\,t\,h\,e\,r,r\,e\,f}+u_{o\,t\,h\,e\,r,r\,e\,f}\left[subj\_pron\left[n\right]\right]+w_{o\,t\,h\,e\,r,r\,e\,f}\left[item\_pron\left[n\right]\right])$$

$$+vtype\left[n\right]\cdot \left(-1\right)\cdot (\beta_{o\,t\,h\,e\,r,i\,n\,t}+u_{o\,t\,h\,e\,r,i\,n\,t}\left[subj\_pron\left[n\right]\right]+w_{o\,t\,h\,e\,r,i\,n\,t}\,\left[i\,t\,e\,m\,\_\,p\,r\,o\,n\,\left[n\right]\right])$$

where:


(15) $$s\,o\,f\,t\,m\,a\,x\,\left(y\right)=e\,x\,p\,\left(y\right)/\sum^{k}\left(y_{k}\right)$$

Then, the probability of the referent NP1 is calculated conditioned on a personal pronoun and on a demonstrative pronoun:


(16) $$P\,\left(N\,P\,1|P\,P,\ldots \right)=\frac{P\,\left(P\,P|N\,P\,1,\ldots \right)}{P\,\left(P\,P|N\,P\,1,\ldots \right)+P\,\left(P\,P|N\,P\,2,\ldots \right)}$$

(17) $$P\,\left(N\,P\,1|D\,P,\ldots \right)=\frac{P\,\left(D\,P|N\,P\,1,\ldots \right)}{P\,\left(D\,P|N\,P\,1,\ldots \right)+P\,\left(D\,P|N\,P\,2,\ldots \right)}$$

These probabilities are used to predict each observation *n* conditional on the type of pronoun that was completed:


(18) $$p\,r\,e\,d_{N\,P\,1_{n}}\,\sim \,B\,e\,r\,n\,o\,u\,l\,l\,i\,\left(P\,\left(r\,e\,f\,e\,r\,e\,n\,t|p\,r\,o\,n\,o\,u\,n_{n},\ldots \right)\right)$$

As before, the … symbolize all the information that the model is taking into account generate the predictions: the characteristics of the stimuli (i.e., intercept, beta, and by-item adjustments) and of the subject performing the free-prompt task (i.e., by-subject adjustments). However, now the pronoun type of each observation affects the predictions of the model.

##### Bayesian Model

The Bayesian model predicts that the probability of referring to *NP1* for pronoun-prompt data is determined by its posterior distribution in the free-prompt data according to Bayes’ rule: the *likelihood* of NP1 (*P(pronoun|referent = NP1*)) is multiplied by the *prior* probability of NP1 (*P(referent = NP1)*), normalized to be a probability distribution by dividing it by the marginal probability distribution of the pronouns. This *posterior* can be estimated by the free-prompt data.

The parameters of the Bayesian model are estimated using equations (9) from the Expectancy model and (11) from the Mirror model. This entails that the model contains the parameters *β_*vtypeNP1*_* and *β_*vtypePP*_*. In addition, since the by-participants and by-items adjustments from both (9) and (11) are used, this model has six potentially correlated by-subject adjustments and three potentially correlated by-items adjustments. For this reason, the parameter estimates are not identical to the previous models. The parameters estimated with the free-prompt data were used to generate predictions for each observation *n* of the pronoun-prompt data as follows.

We calculate the prior *P(NP1)* based on equation (10) and the likelihoods depending on the pronoun type P(*pronoun|NP1*) based on equations (13) and (14). With these we calculate P(*NP1*|*pronoun*).

The posterior probability of the referent NP1 is calculated conditional on a personal pronoun and on a demonstrative pronoun:


(19) $$P\,\left(N\,P\,1|P\,P,\ldots \right)=\frac{P\,\left(P\,P|N\,P\,1\right)\,P\,\left(N\,P\,1\right)}{P\,\left(P\,P|N\,P\,1\right)\,P\,\left(N\,P\,1\right)+P\,\left(P\,P|N\,P\,2\right)\,\left(1-P\,\left(N\,P\,1\right)\right)}$$

(20) $$P\left(NP1|DP,..\right)=\frac{P\,\left(D\,P|N\,P\,1\right)\,P\,\left(N\,P\,1\right)}{P\,\left(D\,P|N\,P\,1\right)\,P\,\left(N\,P\,1\right)+P\,\left(D\,P|N\,P\,2\right)\,\left(1-P\,\left(N\,P\,1\right)\right)}$$

These probabilities are used to predict each observation *n* conditional on the type of pronoun that was completed:


(21) $$p\,r\,e\,d_{N\,P\,1_{n}}\,\sim \,B\,e\,r\,n\,o\,u\,l\,l\,i\,\left(P\,\left(N\,P\,1|p\,r\,o\,n\,o\,u\,n_{n},\ldots \right)\right)$$

#### Parameter Estimates

##### Expectancy Model

Table 1 shows the mean estimate and credible interval for the parameters of the Expectancy model. Applying *logit^–1^* to the parameter values, we estimate the value of *P(NP1)* across verb types, as shown in Table 2.

**TABLE 1:** Mean estimate and credible interval for the parameters of the Expectancy model, Experiment 1.

Parameter	Mean	q5	q95
α_NP1_	–0.68	–0.99	–0.40
β_vtype_	0.30	0.01	0.59

**TABLE 2:** Value of *P*(*NP*1) across verb type for the Expectancy model, Experiment 1.

Variable	Mean	q5	q95
*P*(*NP*1|*verb = accusative*)	0.41	0.33	0.49
*P*(*NP*1|*verb = dative*)	0.28	0.19	0.37

##### Mirror Model

Table 3 shows the mean estimate and credible interval for the parameters of the Mirror model. Applying the *softmax* functions to the parameter values, we estimate the value of *P(NP1)* across verb type and pronoun type, as shown in Table 4.

**TABLE 3:** Mean estimate and credible interval for the parameters of the Mirror model, Experiment 1.

Parameter	Mean	q5	q95
α_*PP*_	2.57	2.01	3.21
α_*other*_	–1.74	–3.00	–0.66
β_*int_PP*_	0.24	–0.22	0.69
β_*int_other*_	–0.35	–1.06	0.37
β_*ref_PP*_	2.24	1.74	2.82
β_*ref_other*_	1.01	0.11	1.87
β_*vtype_PP*_	–0.62	–1.08	–0.16
β_*vtype_other*_	1.05	0.35	1.83

**TABLE 4:** Value of *P*(*NP*1) across verb type and pronoun type for the Mirror model, Experiment 1.

Variable	Mean	q5	q95
*P*(*NP*1|*pronoun = personal, verb = accusative*)	0.75	0.69	0.83
*P*(*NP*1|*pronoun = personal, verb = dative*)	0.57	0.54	0.60
*P*(*NP*1|*pronoun = demonstrative, verb = accusative*)	0.03	0.01	0.06
*P*(*NP*1|*pronoun = demonstrative, verb = dative*)	0.03	0.00	0.08

##### Bayesian Model

Table 5 shows the mean estimate and credible interval for the parameters of the Bayesian model. Applying the *softmax* functions to the parameter values, we estimate the value of *P(NP1)* across verb type and pronoun type, as shown in Table 6.

**TABLE 5:** Mean estimate and credible interval for the parameters of the Bayesian model, Experiment 1.

Parameter	Mean	q5	q95
α_*NP1*_	–0.66	–0.95	–0.38
α_*PP*_	2.54	1.99	3.16
α_*other*_	–1.70	–2.96	–0.58
β_*int_PP*_	0.20	–0.28	0.65
β_*int_other*_	–0.40	–1.12	0.31
β_*ref_PP*_	2.24	1.75	2.79
β_*ref_other*_	1.00	0.09	1.85
β_*vtype_PP*_	–0.62	–1.09	–0.16
β_*vtype_other*_	0.96	0.23	1.75
β_*vtype_NP1*_	0.28	0.00	0.57

**TABLE 6:** Value of *P*(*NP*1) across verb type and pronoun type for the Bayesian model, Experiment 1.

Variable	Mean	q5	q95
*P*(*NP*1|*pronoun = personal, verb = accusative*)	0.79	0.64	0.92
*P*(*NP*1|*pronoun = personal, verb = dative*)	0.22	0.10	0.36
*P*(*NP*1|*pronoun = demonstrative, verb = accusative*)	0.04	0.01	0.10
*P*(*NP*1|*pronoun = demonstrative, verb = dative*)	0.01	0.00	0.02

#### Model Comparison

We compare the models numerically using the expected log-predictive density (elpd) score of the models, with a higher score indicating better predictive accuracy for the held out pronoun-prompt data, as shown in Table 7. There is a clear overall advantage in predictive accuracy for the Bayesian model. When the difference between predictive density (“elpd_diff”) is larger than four and the number of observations is larger than 100, then the normal approximation and the standard errors are quite reliable descriptions of the uncertainty in the difference. As a rule of thumb, differences larger than four are considered enough to differentiate the predictive performance of the models (Sivula et al., 2020). We also calculated the “weight” of the predictions of each model by averaging via stacking of predictive distributions. Stacking maximizes the potential elpd score by pulling the predictions of all the different models together. The values under the weight column represent the relative contribution of each model to the combined optimal model. In this case, the Bayesian model contributes almost 90% to the weighted predictions. In Table 8, we compare just the Mirror and Expectancy models. It is clear that the Mirror model has a predictive performance superior to the Expectancy model.

**TABLE 7:** Model comparison, Experiment 1.

	elpd_diff	se_diff	elpd	se_elpd	weight
Bayesian	0	0	−728	27	0.89
Mirror	−132	14	−860	27	0.00
Expectancy	−238	24	−966	16	0.11

*The table is ordered by the expected log-predictive density (elpd) score of the models, with a higher score indicating better predictive accuracy. The highest scoring model is used as a baseline for the difference in elpd and the difference standard error (SE). “Weight” represents the weights of the individual models that maximize the total elpd score of all the models.*

**TABLE 8:** Comparison of Mirror and Expectancy models.

	elpd_diff	se_diff	elpd	se_elpd	weight
Mirror	0	0	−860	27	0.67
Expectancy	−106	31	−966	16	0.33

*The table is ordered by the expected log-predictive density (elpd) score of the models, with a higher score indicating better predictive accuracy. The highest scoring model is used as a baseline for the difference in elpd and the difference standard error (SE). “Weight” represents the weights of the individual models that maximize the total elpd score of all the models.*

In Table 9, we show the difference in predictive density for the models split by verb type and pronoun type.

**TABLE 9:** Difference in expected log-predictive density (elpd_diff) of the models assessed for four subsets of the data from Experiment 1, depending whether the verb is accusative or dative, and whether the pronoun shown is personal (PP) or demonstrative (DP).

Model	elpd_diff	se_diff	weight
**PP – accusative**			
Bayesian	0.0	0.0	0.11
Mirror	–11.1	6.5	0.63
Expectancy	–56.8	11.9	0.26
**DP – accusative**			
Bayesian	0.0	0.0	1.00
Mirror	–2.5	1.7	0.00
Expectancy	–136.7	11.2	0.00
**PP – dative**			
Bayesian	0.0	0.0	0.48
Mirror	–113.9	10.0	0.00
Expectancy	0.2	4.2	0.52
**DP – dative**			
Bayesian	0.0	0.0	0.76
Mirror	–4.1	5.7	0.00
Expectancy	–44.3	16.8	0.24

**FIGURE 4:**
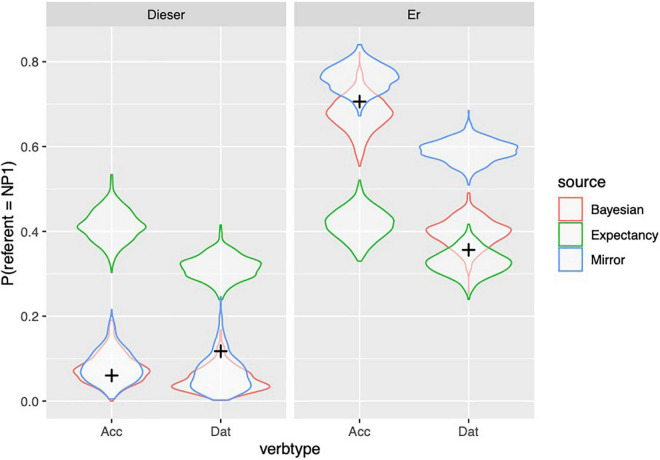
Observed proportion of responses (from held out data, Experiment 1) are depicted with black crosses; distribution of simulated proportions based on the model predictions are depicted with violin plots.

Figure 4 shows to what extent the predictions of the different models, depicted with violin plots, match the observed held out data from the participants. The predictions of the models are shown by means of their posterior predictive distribution: simulated datasets generated based on the posterior distributions of its parameters. The posterior predictive distribution shows what other possible datasets may look like. Because we show held-out data (in contrast with data used to “train” the model), we can compare the three models based on the extent to which the held out data looks more plausible under the predictive distributions. By-participant and by-item predictions of the models are depicted in Supplementary Figures 1, 2 which can be found in the Supplementary Material.

From Figure 4 and Table 9, we can see that the observed data are within the distribution of predictions of the Bayesian model in every condition, whereas the data cannot be accounted by the other models under all conditions. However, the Bayesian model is only clearly superior to the Mirror model for the personal pronoun in the dative contexts (while the Expectancy model performs much better here than it does in other conditions). The Mirror model comes close to the performance of the Bayesian model in the other three conditions, even though the Bayesian model is numerically superior.

#### Evaluating the Strong Form of the Bayesian Model

Here, we evaluate the claims of the strong form of the Bayesian model. First, to examine the influence of verb type on the prior and the production likelihoods, a model comparison was carried out comparing models with and without verb type in the prior and in the pronoun production likelihoods to assess the impact on predictive accuracy of the resulting models. Tables 10, 11 show the outcome of the model comparison.

**TABLE 10:** Model comparisons after removing verb type from the likelihood and the prior.

	elpd_diff	se_diff	elpd	se_elpd	weight
Full Bayesian	0	0.0	−728	27	0.97
No verb type in likelihood	−24	7.0	−753	28	0.03
No verb type in prior	−55	7.5	−783	27	0.00

*The table is ordered by the expected log-predictive density (elpd) score of the models, with a higher score indicating better predictive accuracy. The highest scoring model is used as a baseline for the difference in elpd (elpd_diff) and the difference standard error (se_diff). ‘Weight’ represents the weights of the individual models that maximize the total elpd score of all the models.*

**TABLE 11:** Model with no verb type in the likelihood compared to a model with no verb type on the prior.

	elpd_diff	se_diff	elpd	se_elpd	weight
No verb type in likelihood	0	0.0	−753	28	0.87
No verb type in prior	−30	9.2	−783	27	0.13

*The table is ordered by the expected log-predictive density (elpd) score of the models, with a higher score indicating better predictive accuracy. The highest scoring model is used as a baseline for the difference in elpd (elpd_diff) and the difference standard error (se_diff). “Weight” represents the weights of the individual models that maximize the total elpd score of all the models.*

The model comparison shows that the verb type has a large impact for the predictions of the model, and that the predictions of the Bayesian model deteriorate the most when the verb type information is removed from the prior. A model without verb type on the prior performs significantly worse than a full model (Table 10) and a model without verb type on the production likelihood (Table 11), demonstrating that the prior is influenced by verb type information, which is in line with the strong form of the Bayesian model. But removing verb type from the production likelihood also has a detrimental impact on predictive accuracy when compared to a full model. To explore this in more detail, we examine the influence of verb type on likelihoods for the personal and demonstrative pronouns separately.

We ran Bayesian multilevel models with the sum-coded factors Referent (proto-agent/NP1 versus proto-patient/NP2) and Verb Type (accusative versus dative) with random intercepts for participants and items, using the brms package (Bürkner, 2017) in RStudio (RStudio Team, 2019) on R version 3.6.1 (R Core Team, 2019)^13^. For the demonstrative pronouns, there was a clear effect of Referent (mean estimate −1.74, 95% CrI −2.24, −1.31), no effect of Verb Type (mean estimate 0.33, 95% CrI −0.11, 0.80) and no interaction between the two factors (mean estimate −0.12, 95% CrI −0.56, 0.34). This can be interpreted as follows: participants used a demonstrative pronoun to refer to the proto-patient (NP2) much more often than when referring to the proto-agent (NP1), across both accusative and dative verbs. For the personal pronouns, the model showed a clear effect of Referent in the opposite direction (mean estimate 1.46, 95% CrI 1.18, 1.78). The model also showed an effect for Verb Type, but the estimate here was closer to zero (mean estimate −0.75, 95% CrI −1.04, −0.45). There was no interaction between Referent and Verb Type (mean estimate 0.24, 95% CrI −0.03, 0.52). This shows that participants used a personal pronoun to refer to the proto-agent (NP1) more often than when referring to the proto-patient (NP2). The overall rate of pronominalization for the personal pronoun was higher for the dative verbs compared to the accusative verbs, but the relative (NP1–NP2) production bias was not influenced by verb type.

### Discussion

In Experiment 1, the Bayesian model clearly outperforms both the Mirror model and Expectancy model overall. Additionally, the model is able to account better for both the personal and demonstrative pronouns than the competing models when performance is assessed separately for each pronoun in all but two comparisons, although the degree of difference between models does vary (see Table 9)^14^. The Mirror model comes close to the performance of the Bayesian model in two out of four conditions, and for demonstratives in the accusative contexts Bayesian and Mirror model performance is indistinguishable. The Expectancy model is outperformed by both the Mirror and the Bayesian models except for personal pronouns in dative contexts, where Bayesian and Expectancy are indistinguishable and both far outperform the Mirror model. The variation over the different conditions demonstrates, however, that the Bayesian model is more powerful for taking into account elements of both other models, i.e., movement in the prior (Expectancy) and production likelihoods (Mirror), while neither element alone can capture behavior across the conditions.

We also tested the predictions of the strong Bayesian model. In our analysis, verb type had a larger influence on the prior than on the likelihoods, which is in line with the strong Bayesian model. But removing verb type from the likelihood also had negative impact on predictive accuracy. However, given that the verb type contrast in this experiment encompasses a change in position of the subject (NP1 in accusative verbs and NP2 in dative verbs), the constructions are perhaps not directly comparable.

The second test of the strong Bayesian model was examining the pattern of results in the pronoun production likelihoods separately for personal and demonstrative pronouns. Here we saw no interaction of verb type with referent; this is in line with the strong Bayesian model which states that likelihoods should not be influenced by verb type, although it should be noted that the verb type under examination here is of a different nature than the verb contrasts normally examined. Additionally, we were interested in the relative influence of subjecthood and agentivity, because the two factors make contrasting predictions for the effect of Referent (NP1 versus NP2) across the two verb types. In previous studies, subjecthood (and/or topichood) influenced production likelihoods. Our results were as follows: demonstrative pronouns were much more likely to be produced when referring to NP2 versus NP1 across both verb types. The NP2 was the proto-patient in both accusative and dative verbs, suggesting a strong influence of non-agentivity rather than non-subjecthood. Personal pronoun likelihoods, on the other hand, showed a less clear pattern. For the accusative verbs there was a clear NP1 (subject/proto-agent) advantage. There was a weaker advantage for NP1 (object/proto-agent) in the dative verbs, but the difference in NP1 advantage was not confirmed statistically. Overall participants were more likely to produce a pronoun following dative verbs compared to accusative verbs. The proto-agent advantage speaks for an influence of agentivity rather than subjecthood, but the pattern in the dative verbs is nevertheless puzzling and prevents us from drawing strong conclusions here.

It is certainly the case that the verb type contrast examined here (accusative versus dative verbs) is of a different nature than the contrasts tested previously. While an IC contrast, exemplified in (5) and (6), represents a difference in expected continuations, the accusative–dative contrast represents a difference in the assignment of argument roles. It is therefore perhaps not surprising that the patterns in Experiment 1 are different from previous studies in which an IC contrast was used. For this reason, we carried out Experiment 2, using an IC contrast to make our results more comparable to previous studies. This experiment also gives us a chance to replicate our findings with respect to overall model performance and represents a more straightforward test of the predictions of the strong Bayesian model.

## Experiment 2

Experiment 2 was a text continuation task with the German personal pronoun *er* and the demonstrative pronoun *dieser* using an IC-based verb-type contrast, more closely reflecting materials in previous studies (Kehler et al., 2008; Rohde and Kehler, 2014). Specifically, we used stimulus–experiencer (SE) and experiencer–stimulus (ES) verbs (see Bott and Solstad, 2014 for an overview of the semantic properties of these verbs). In addition, the contrast allows us to look again at the contribution of agentivity and subjecthood. Recall that we pursue the proto-role approach (Dowty, 1991), where thematic roles are characterized by features associated with proto-agents and proto-patients. Experiencers are typically considered agent-like because they entail sentience.

In ES constructions, subjects and experiencers (as the highest thematic role) are aligned, potentially yielding a higher production likelihood for NP1. In SE constructions, NP1 outranks NP2 with respect to subjecthood but NP2 outranks NP1 with respect to agentivity^15^. A subset of dative items from Experiment 1 were also included in an attempt to verify the pattern in the production likelihoods from Experiment 1^16^.

### Participants

Forty participants (18–67 years) were recruited on the online platform Prolific.ac to take part in Experiment 2. Data from all 40 participants (15 female, 25 male) were used in the analysis. All participants indicated that they were German native speakers; 8 participants were bilingual. No participants reported language-related disorders. All participants gave their consent and received a small fee for participation.

### Materials

Thirty six critical items were constructed, 18 SE items and 18 ES items. 28 verbs were taken from Bott and Solstad (2014) who systematically tested the semantics of implicit causality verbs in a set of German verbs; additional verbs were pretested according to the “*that*-clause replacement test” and the “*absichtlich*-test” (adverbial “*deliberately*” being added to transitive verb frames) following Bott and Solstad (2014). In order to avoid effects of polarity, each of the two groups of 18 critical items included nine verbs related to negative perception (e.g., *schockieren* “shock,” SE; *verachten* “despise,” ES) and nine that were positive (e.g., *faszinieren* “fascinate,” SE; *respektieren* “respect,” ES). The critical items consisted of a context sentence which contained a nominative argument, the main verb in present tense and an accusative argument, and a prompt sentence which was either a personal pronoun-prompt (*er*), a demonstrative pronoun-prompt (*dieser*) or a free-prompt (blank line). In both SE and ES items, contexts were presented in canonical order (subject verb object). Example items are given in (22) and (23).

(22) SE items:

(a) *Er prompt:* Der Jurist faszinierte den Richter. Er _________
(b) *Dieser prompt:* Der Jurist faszinierte den Richter. Dieser _________
(c) *Free-prompt:* Der Jurist faszinierte den Richter. _________

“The lawyer *(nom.masc.)* fascinated the judge (*acc.masc.*). *He/DEM/*…”

(23) ES items:

(a) *Er prompt:* Der Christ respektierte den Moslem. Er _________
(b) *Dieser prompt:* Der Christ respektierte den Moslem. Dieser _________
(c) *Free-prompt:* Der Christ respektierte den Moslem. _________

“The Christian *(nom.masc.)* respected the Muslim (*acc.masc.*). *He/DEM/*…”

For both NPs, role names were chosen following the same criteria as in Experiment 1. 22 filler items were also created: six dative-experiencer contexts from Experiment 1, four nominative-accusative IC verb contexts followed by a connector, three contexts with a single NP followed by a connector, seven catch fillers (included to ensure that participants were paying attention to the task), and two *dieser* items to prime a pronominal reading (see Experiment 1 for a description). The filler set included a mix of feminine and masculine pronouns and referents to counterbalance the large number of masculine referents in the critical items. The items were distributed over three lists in a Latin-square design.

### Procedure

Based on a short description of the task, participants could choose to take part in the study via the Prolific.ac application. Participants gave their consent and answered a short series of biographical questions before starting the experimental task. Task instructions were the same as for Experiment 1.

### Data Coding

Data was coded in the same way as for Experiment 1. The two annotators agreed in 86% of observations, with a Cohen’s (unweighted) Kappa of 0.78 (*z* = 45.5, *p* < 0.0001). Data from 40 participants for 12 items (6 SE, 6 ES) per prompt condition resulted in a total of 1440 observations; 480 each in the *er*-prompt, *dieser*-prompt and free-prompt conditions. The distribution of reference and the response categories for the first referential expression are given in Supplementary Material. For the following analyses, the dataset was reduced in the same way as in Experiment 1, leaving a total of 1221 observations for the analysis (352 free-prompt, 430 *dieser*-prompt and 439 *er*-prompt).

### Data Analysis

A data analysis plan and accompanying predictions were registered in advance of carrying out this experiment on aspredicted.org. The registration can be found in Supplementary Material. While the data collection followed the registered plan, the data analysis was in the end superseded by the Bayesian statistical analysis presented here. This type of analysis was a late addition to the project that we did not foresee at the time of data collection. Data analysis and models are the same as in Experiment 1, with the exception that the verb types are ES (coded as 1) and SE (coded as −1).

### Results

**FIGURE 5:**
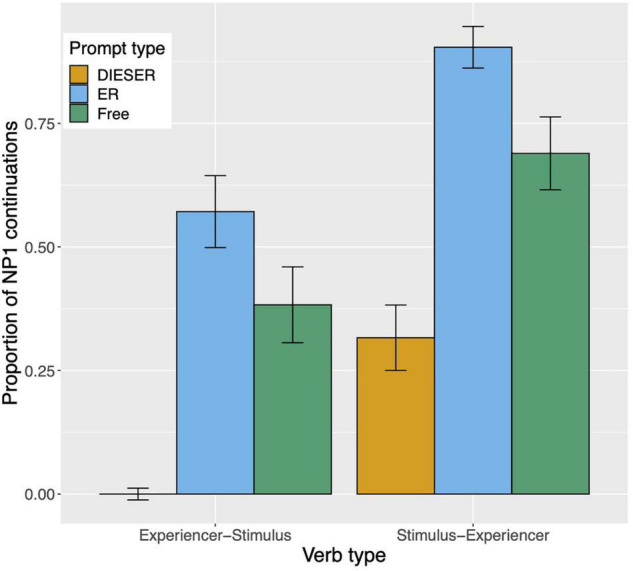
Proportion of NP1 continuations per prompt condition (*er, dieser, and free*) and verb type in Experiment 2. The NP1 continuations represent the next-mention bias for NP1. Error bars represent 95% confidence intervals based on normalized data following Morey (2008) and Chang (2013).

**FIGURE 6:**
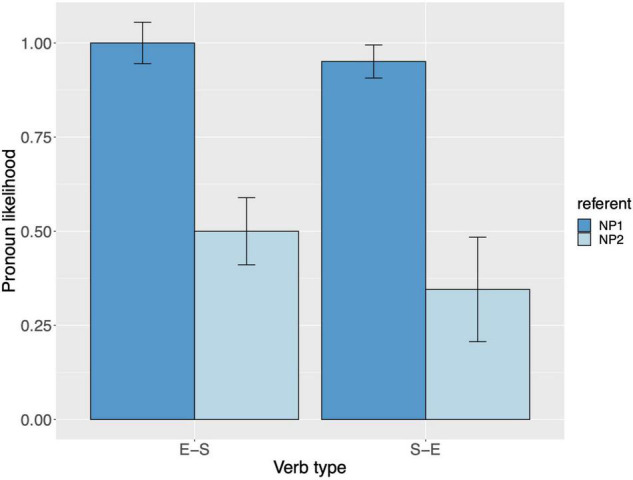
Personal pronoun: probability of using a personal pronoun to refer to NP1 and NP2, per verb type, for Experiment 2. These bars represent the pronoun production likelihoods (and are therefore based on the free-prompt data). Error bars are by-participant and represent 95% confidence intervals based on normalized data (Morey, 2008; Chang, 2013).

**FIGURE 7:**
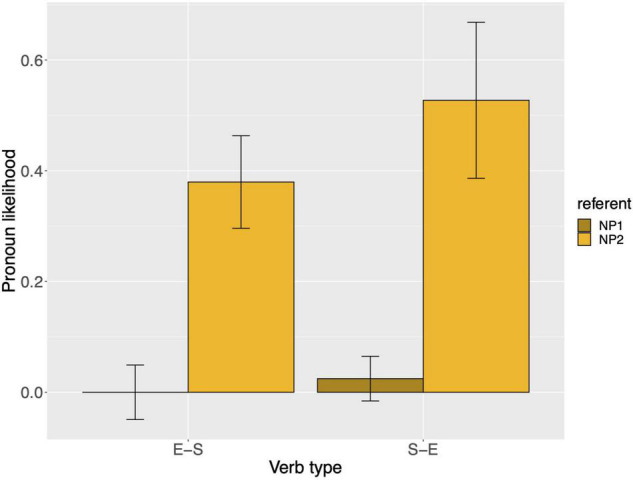
Demonstrative pronoun: probability of using a demonstrative pronoun to refer to NP1 and NP2, per verb type, for Experiment 2. These bars represent the pronoun production likelihoods (and are therefore based on the free-prompt data). Error bars are by-participant and represent 95% confidence intervals based on normalized data (Morey, 2008; Chang, 2013).

Raw proportions for the next-mention bias are shown in Figure 5. Figure 6 shows the personal pronoun production likelihoods, and Figure 7 the demonstrative pronoun production likelihoods^17^. When calculating likelihoods for the personal pronoun, both subject and non-subject personal pronouns were included. For the demonstrative pronoun, both subject and non-subject demonstrative pronoun *dieser*, and subject and non-subject demonstrative pronoun *der*, were included.

A follow-up rating experiment was also conducted: see discussion below. Method and results for this rating experiment can be found in Supplementary Material.

#### Parameter Estimates

##### Expectancy Model

Table 12 shows the mean estimate and credible interval for the parameters of the Expectancy model. Applying *logit^–1^* to the parameter values, we estimate the value of *P(NP1)* across verb types, as shown in Table 13.

**TABLE 12:** Mean estimate and credible interval for the parameters of the Expectancy model, Experiment 2.

Parameter	Mean	q5	q95
α_NP1_	0.19	–0.29	0.66
β_vtype_	–0.94	–1.51	–0.42

**TABLE 13:** Value of *P*(*NP*1) across verb type for the Expectancy model, Experiment 2.

Variable	Mean	q5	q95
*P*(*NP*1|*verb = ES*)	0.33	0.18	0.48
*P*(*NP*1|*verb = SE*)	0.75	0.61	0.87

##### Mirror Model

Table 14 shows the mean estimate and credible interval for the parameters of the Mirror model. Applying *softmax* to the parameter values, we estimate the value of *P(NP1)* across verb type and pronoun type, as shown in Table 15.

**TABLE 14:** Mean estimate and credible interval for the parameters of the Mirror model, Experiment 2.

Parameter	Mean	q5	q95
α_*PP*_	3.67	2.51	5.0
α_*other*_	–3.46	–5.39	–1.6
β_*int_PP*_	0.55	–0.41	1.6
β_*int_other*_	–0.47	–2.11	1.1
β_*ref_PP*_	3.68	2.61	4.9
β_*ref_other*_	–0.04	–1.69	1.5
β_*vtype_PP*_	1.00	0.04	2.1
β_*vtype_other*_	0.08	–1.44	1.6

**TABLE 15:** Value of *P*(*NP*1) across verb type and pronoun type for the Mirror model, Experiment 2.

Variable	Mean	q5	q95
*P*(*NP*1|*pronoun = personal, verb = ES*)	0.64	0.56	0.74
*P*(*NP*1|*pronoun = personal, verb = SE*)	0.73	0.60	0.87
*P*(*NP*1|*pronoun = demonstrative, verb = ES*)	0.00	0.00	0.01
*P*(*NP*1|*pronoun = demonstrative, verb = SE*)	0.01	0.00	0.03

##### Bayesian Model

Table 16 shows the mean estimate and credible interval for the parameters of the Bayesian model. Applying *softmax* to the parameter values, we estimate the value of *P(NP1)* across verb type and pronoun type, as shown in Table 17.

**TABLE 16:** Mean estimate and credible interval for the parameters of the Bayesian model, Experiment 2.

Parameter	Mean	q5	q95
α_*NP1*_	0.17	–0.30	0.66
α_*PP*_	3.60	2.51	4.82
α_*other*_	–3.41	–5.39	–1.58
β_*int_PP*_	0.56	–0.39	1.62
β_*int_other*_	–0.52	–2.07	0.96
β_*ref_PP*_	3.61	2.58	4.78
β_*ref_other*_	–0.07	–1.74	1.49
β_*vtype_PP*_	0.96	0.03	2.00
β_*vtype_other*_	0.06	–1.45	1.59
β_*vtype_NP1*_	–0.94	–1.50	–0.42

**TABLE 17:** Value of *P*(*NP*1) across verb type and pronoun type for the Bayesian model, Experiment 2.

Variable	Mean	q5	q95
*P*(*NP*1|*pronoun = personal, verb = ES*)	0.67	0.31	0.92
*P*(*NP*1|*pronoun = personal, verb = SE*)	0.71	0.35	0.95
*P*(*NP*1|*pronoun = demonstrative, verb = ES*)	0.00	0.00	0.01
*P*(*NP*1|*pronoun = demonstrative, verb = SE*)	0.02	0.00	0.06

#### Model Comparison

As before, we compare the models numerically using the elpd score of the models, as shown in Table 18. In Table 19 we show the elpd score of the models split by verb type and pronoun type. There is again a clear overall advantage in predictive accuracy for the Bayesian model (Table 18). The Bayesian model contributes 90% to the weighted predictions in the overall comparison.

**TABLE 18:** Model comparison, Experiment 2.

	elpd_diff	se_diff	elpd	se_elpd	weight
Bayesian	0	0	-368	19	0.9
Mirror	-98	13	-467	24	0.0
Expectancy	-209	23	-578	16	0.1

*The table is ordered by the expected log-predictive density (elpd) score of the models, with a higher score indicating better predictive accuracy. The highest scoring model is used as a baseline for the difference in elpd (elpd_diff) and the difference standard error (se_diff). “Weight” represents the weights of the individual models that maximize the total elpd score of all the models.*

**TABLE 19:** Difference in expected log-predictive density (elpd_diff) of the models assessed for four subsets of the data from Experiment 2, depending whether the verb is stimulus–experiencer (SE) or experiencer–stimulus (ES), and whether the pronoun shown is personal (PP) or demonstrative (DP).

Model	elpd_diff	se_diff	weight
**PP – SE**			
Bayesian	0.00	0.00	1.00
Mirror	–25.92	3.86	0.00
Expectancy	–25.91	5.15	0.00
**DP – SE**			
Bayesian	0.00	0.00	0.69
Mirror	–48.74	8.12	0.00
Expectancy	–53.10	19.28	0.31
**PP – ES**			
Bayesian	0.00	0.00	0.37
Mirror	–23.84	8.40	0.28
Expectancy	–14.24	7.34	0.35
**DP – ES**			
Bayesian	0.00	0.00	0.19
Mirror	0.06	0.45	0.81
Expectancy	–115.91	6.24	0.00

**FIGURE 8:**
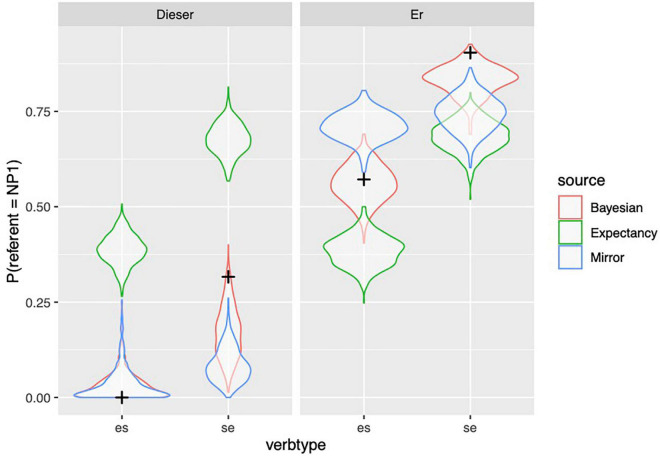
Observed proportion of responses (from held out data, Experiment 2) are depicted with black crosses; distribution of simulated proportions based on the model predictions are depicted with violin plots.

Figure 8 shows to what extent the predictions of the different models, depicted with violin plots, match the observed held out data from the participants, as per Experiment 1. By-participant and by-item predictions of the models are depicted in Supplementary Figures 3, 4 which can be found in the Supplementary Material.

From Figure 8 and Table 19, it is clear that the observed data are well within the distribution of predictions of the Bayesian model, whereas the data cannot be accounted by the other models under all conditions. Unlike in Experiment 1, here the Bayesian model outperforms the Mirror model in all conditions except for demonstrative pronouns in the ES contexts, where performance of the two models is indistinguishable. The Bayesian model outperforms the Expectancy model in all conditions.

#### Evaluating the Strong Form of the Bayesian Model

Here, we evaluate the claims of the strong form of the Bayesian model by again examining (i) the influence of verb type on the prior (i.e., the next-mention bias) and (ii) the influence of verb type and the relative contribution of agentivity and subjecthood on the pronoun production likelihoods (i.e., on P(pronoun|referent)). First, to examine the influence of verb type, a model comparison was carried out comparing models with and without verb type in the prior and in the pronoun production likelihoods to assess the impact on predictive accuracy of the resulting models. Tables 20, 21 show the outcome of the model comparison.

The model comparison shows that the verb type has a large impact for the predictions of the model, and that the predictions of the Bayesian model deteriorate the most when the verb type information is removed from the prior. A model without verb type on the prior performs significantly worse than a full model (Table 20) and worse than a model without verb type on the production likelihood (Table 21), demonstrating that the prior is influenced by verb type information, in line with the strong form of the Bayesian model. Removing verb type from the production likelihood also has a detrimental impact on predictive accuracy when compared to a full model, demonstrating that overall production likelihoods are also to some extent influenced by verb type; this is explored further by examining the factors affecting the production likelihoods for personal and demonstrative pronouns separately. This was done using Bayesian multilevel models with the same set up as in Experiment 1.

For the demonstrative pronouns, there was a clear effect of Referent (mean estimate −2.37, 95% CrI −3.13, −1.71), no effect of Verb Type (mean estimate −0.49, 95% CrI −1.22, 0.17) and no interaction between the two factors (mean estimate −0.18, 95% CrI −0.89, 0.45). This shows that participants used a demonstrative pronoun to refer to the object (NP2) much more often than when referring to the subject (NP1), across both SE and ES verbs. For the personal pronouns, the model showed a clear effect of Referent (mean estimate 2.35, 95% CrI 1.77, 3.05). The model also showed an effect for Verb Type, but the lower bound of the Credible Interval is almost at zero (mean estimate 0.62, 95% CrI 0.06, 1.27). There was no interaction between Referent and Verb Type (mean estimate 0.35, 95% CrI −0.23, 1.00). This shows that participants used a personal pronoun to refer to the subject (NP1) more often than when referring to the object (NP2), regardless of verb type. The overall rate of pronominalization for the personal pronoun may be slightly higher for the ES verbs compared to the SE verbs, but this effect should be interpreted with caution. We discuss the implications for the relative contributions of subjecthood and agentivity below.

### Discussion

In Experiment 2 the Bayesian model again clearly outperforms both the Mirror model and Expectancy model overall. The Bayesian model is able to account better for both the personal and demonstrative pronouns than the competing models when performance is assessed separately for each pronoun (see Table 19), although the degree of difference between models does vary as before. The Bayesian model outperforms the Mirror model in three out of four conditions. The caveat about the Expectancy model predictions for the demonstrative still applies, but again the Bayesian model outperforms the Expectancy model for the personal pronouns.

One surprising pattern in Experiment 2 is the high number of NP1 continuations with the *dieser* prompt for the SE verbs (see Figure 5). The Bayesian model does a good job of predicting this pattern, although the predicted values are spread out, indicating less certainty about the prediction (see Figure 8). Nevertheless, the high number of NP1 interpretations here is not expected, given the more rigid tendencies of demonstratives. We suspected that this could be due to the experimental design: SE contexts strongly bias toward continuations about the stimulus subject (NP1). At the same time, demonstratives would normally avoid reference to a subject. As such, being presented with a *dieser* prompt in SE contexts presents something of a challenge to participants who may be conflicted about continuing with a less preferred referent (experiencer in this case) but working with the *dieser* bias, or working against the *dieser* bias but satisfying the bias to talk about the stimulus. In order to check whether our suspicion was correct, we carried out a follow-up rating experiment which is described in Supplementary Material. We predicted that the completions in which *dieser* refers to NP1 in the SE condition should be less felicitous than SE completions in which *dieser* refers to NP2, since only the latter works with the grammatical bias associated with *dieser*, and less felicitous than SE completions in which *er* refers to NP1, because *er* does not have a bias against NP1 reference. Our predictions were borne out; a cumulative link model showed that both *dieser*–NP2 completions and the *er*–NP1 completions were significantly more likely to elicit better ratings than *dieser*–NP1 completions

(*z* = 11.52 for *dieser*--NP2 and 12.28 for *er*--NP1)^18^. Given this result, it is striking that the Bayesian model is able to capture the actual data from the SE *dieser–*NP1 completions, and at the same time reflect the uncertainty about the predictions in this condition, which is also reflected in the rating data from the follow-up experiment.

Finally, we again tested the predictions of the strong form of the Bayesian model. As in Experiment 1, the model comparisons for Experiment 2 showed that removing verb type from the prior had a more detrimental effect on the predictive accuracy of the model than removing it from the likelihood, underlining the influence of verb type on prior as found by Rohde and Kehler (2014). While predictive accuracy was also affected by removing verb type from the likelihoods, there was no Verb Type by Referent interaction when the likelihoods were examined separately for each pronoun; this finding provides further support for the strong Bayesian model.

Turning to the relative influence of subjecthood and agentivity on the likelihoods, this was again tested via an effect of Referent. Here, we saw strong effects for personal and demonstrative pronouns, in opposite directions^19^. The pattern shows a strong influence of subjecthood for personal pronouns and an objecthood bias in the likelihoods for demonstrative pronouns, regardless of the thematic role of the subjects and objects. We return to these findings in the general discussion.

## General Discussion

In this study we set out to test the following questions:

• Which model for pronouns (Bayesian, Expectancy or Mirror) best accounts for the interpretation of German personal and demonstrative pronouns?
• Is the resolution of demonstratives as rigid as some previous studies suggest, or is the interpretation influenced by the next-mention bias, as the Bayesian model would predict?
• Is there evidence for the strong form of the Bayesian model?

We evaluated overall model performance by using data from the free-prompt conditions in two text-continuation experiments to generate predictions for the Bayesian, Expectancy and Mirror models. These predictions were compared to actual interpretations from the pronoun-prompt conditions, allowing us to assess the predictive accuracy of the models. Overall results from Experiments 1 and 2 show convincingly that the Bayesian model outperforms both the Expectancy and the Mirror models. When the performance was evaluated per verb type and pronoun type separately, the Mirror model performed almost as well as the Bayesian model in three conditions of Experiment 1 but not in Experiment 2, where the Bayesian model outperformed the Mirror model in three out of four conditions. The Bayesian model was even able to predict behavior that was somewhat unexpected, as in the higher-than-expected number of interpretations of *dieser* as NP1 in the SE condition. The fact that the Bayesian model outperforms the Mirror and Expectancy models is further confirmation of the findings from Rohde and Kehler (2014) and Zhan et al. (2020), and in fact the model performance of the Bayesian model as evaluated here (in particular for Experiment 2) is actually better than in those studies, where the Bayesian and Mirror models showed a similar performance in certain conditions. This validates the approach in Kehler et al. (2008) and Kehler and Rohde (2013) of applying simple Bayesian principles to the complex problem of pronoun resolution.

The fact that the Mirror model was more competitive with the Bayesian model in Experiment 1 than Experiment 2 can be attributed to the verb type contrasts under investigation. The IC contrast in Experiment 2 represents a difference in expected continuations, i.e., a contrast in the prior. Given that the Mirror model does not include the prior, it is not surprising that it does not capture all the data here. On the other hand, the accusative–dative contrast in Experiment 1 represents a difference in the assignment of argument roles, which does not entail such extreme movement of the prior, allowing the Mirror model to perform better. Nonetheless, the overall performance of the Mirror model in Experiment 1 was not as good as the performance of the Bayesian model.

The three models were implemented for the first time in a Bayesian statistical framework, which goes beyond the modeling in previous studies (Rohde and Kehler, 2014; Bader and Portele, 2019; Zhan et al., 2020) and has a number of advantages. The fully hierarchical structure allowed us to accommodate participant and item effects directly into our predictions without averaging. In contrast to previous evaluations of model performance, the Bayesian statistical approach allows us to estimate the parameters of a distribution of predicted observations, and as such we can make more stable inferences about model performance. In the Bayesian statistical approach there is no requirement for additive smoothing (as in Zhan et al., 2020) because the uniform Beta prior over the intercept ensures that probability estimates cannot be zero or one; this is especially important for the demonstrative pronouns where the less flexible interpretation leads to zeros in some cells of the design. Our modeling approach also allowed us to evaluate claims about the strong form of the Bayesian model in a new way, by removing verb type from model components and evaluating the impact on the predictive accuracy of the models. This revealed that removing verb type from the prior had a detrimental impact on the predictive accuracy of the model, which is in line with the strong Bayesian model.

**TABLE 20:** Model comparisons after removing verb type from the likelihood and the prior for Experiment 2.

	elpd_diff	se_diff	elpd	se_elpd	weight
Full Bayesian	0	0.0	−368	19	1
No verb type in likelihood	−35	6.5	−404	23	0
No verb type in prior	−61	9.0	−429	22	0

*The table is ordered by the expected log-predictive density (elpd) score of the models, with a higher score indicating better predictive accuracy. The highest scoring model is used as a baseline for the difference in elpd (elpd_diff) and the difference standard error (se_diff). “Weight” represents the weights of the individual models that maximize the total elpd score of all the models.*

**TABLE 21:** Model with no verb type in the likelihood compared to a model with no verb type on the prior, Experiment 2.

	elpd_diff	se_diff	elpd	se_elpd	weight
No verb type in likelihood	0	0	−404	23	0.82
No verb type in prior	−25	9	−429	22	0.18

*The table is ordered by the expected log-predictive density (elpd) score of the models, with a higher score indicating better predictive accuracy. The highest scoring model is used as a baseline for the difference in elpd (elpd_diff) and the difference standard error (se_diff). “Weight” represents the weights of the individual models that maximize the total elpd score of all the models.*

In this study we examined German personal and demonstrative pronouns, a new contribution to the evidence about the Bayesian model for pronouns. It also provides a new perspective on the interpretation of demonstrative pronouns. German demonstratives have been long neglected in literature on pronoun resolution, and have only recently gained more attention. Here too the main debate has focussed on identifying particular factors that influence resolution and that can distinguish preferences for the resolution of demonstratives from those of personal pronouns. The current study may shift the debate toward understanding the resolution of demonstratives in the context of a speaker and an addressee, where predictions about the message content are combined with the estimation of the speaker’s choice of referential form.

It was noted that the Expectancy model (i.e., our operationalization of Expectancy as P(referent)) was not predicted to capture demonstrative pronouns because of their tendency to refer to less “expected” referents. Indeed, the modeling results for Expectancy model in the demonstratives confirm that this approach is not successful. The Bayesian model, too, makes use of expectations about the upcoming referent (i.e., the next-mention bias), and it was possible therefore that the Bayesian model would not be so successful for demonstratives. But our results show that this is not the case. The combination of next-mention bias with production likelihoods is a powerful modification that makes the Bayesian model flexible enough to accommodate pronoun types with quite different resolution tendencies. As such, our study has shown that the Bayesian model is not limited to just one type of pronoun.

That being said, broad cross-linguistic evidence for the Bayesian model is lacking, having previously been evaluated fully only on flexible personal pronouns in English and pronouns in Mandarin Chinese (where null and overt pronouns appear to overlap in resolution preferences). While the current study allows us to incorporate demonstrative pronouns into the Bayesian model without revising its basic assumptions, it remains to be seen whether this is the case for a wider variety of pronouns or indeed other types of anaphora. A broader exploration of pronoun systems and languages would therefore be welcomed, as well as studies presenting more than two potential referents for a pronoun.

In addition to the modeling outcomes, the current study reveals some general patterns in the resolution of *dieser*, which has not been extensively empirically tested. In Experiment 1 the *dieser* prompt showed a strong resolution to the NP2/proto-patient, even when the proto-patient was a subject as in the dative contexts. In Experiment 2, conversely, *dieser* was resolved exclusively to the NP2/object in the ES conditions and showed a tendency to the NP2/object in the SE conditions. Taking both experiments together, *dieser* appears not to follow an anti-subject bias nor an anti-agent bias, contra several claims in the literature (Fuchs and Schumacher, 2020; Patil et al., 2020). The overall pattern is a strong preference to refer to the NP2, regardless of grammatical or thematic role. Indeed, the follow-up experiment to Experiment 2 showed that resolving *dieser* to NP1 was less felicitous than resolving to NP2, and less felicitous than resolving *er* to NP1, underlining a preference for the second-mentioned referent (at least in the limited set of contexts presented in our study). But the contrast in interpretations for *dieser* between ES and SE contexts does point to the interpretation being affected by the next-mention bias. However, the outcome of the follow-up experiment also underlines the challenge of testing pronouns with less flexible interpretation preferences: this can create conflict in some conditions when context biases and pronoun biases clash, leading to productions and/or interpretations that would not normally be considered by participants.

Finally, in this study we attempted to assess the relative contribution of agentivity and subjecthood to pronoun production likelihoods. The strong form of the Bayesian model claims that likelihoods should be affected by subjecthood (and/or topichood); studies of German pronouns have shown that agentivity is important for interpretation, but until now it has not been demonstrated whether agentivity influences expectations about an upcoming referent or acts on the likelihood of producing a pronoun. The pattern for production likelihoods in Experiment 1 revealed an agentivity influence on likelihoods (proto-agents for personal pronouns and proto-patients for demonstratives), but the pattern for personal pronouns was unclear. Particularly striking were the production biases seen in dative contexts, where personal pronouns were the preferred referential forms for both potential referents – an effect not seen in previous studies. Whereas previous work has argued that subjecthood leads to a strong pronominalization bias, this study is the first to show that this bias applies to subjects that are not NP1 in argument structure. In Experiment 2 the production likelihoods were only affected by grammatical role (personal pronouns produced for subjects, demonstratives for objects) and there was no evidence of agentivity having an influence. It should be noted that the contrast in agentivity features between experiencers and stimuli (i.e., between NP1 and NP2 in SE and ES contexts) is not very large, certainly not as clear as the contrast between proto-agents and proto-patients in accusative and dative verbs. Some research has suggested that in SE contexts the stimulus is more “agent-like” than the experiencer (Dowty, 1991). This could have led to the agentivity influence not being detectable in Experiment 2. However, having the two factors, grammatical role and agentivity, being manipulated via verb type makes it harder to interpret the claims about a lack of verb type influence on likelihoods, as would be predicted under the strong form of the Bayesian model. Overall, the results from both experiments make it difficult to draw firm conclusions about the relative contributions of these factors. This aspect should be tested further in future experiments with an altered design.

Our study makes the following contributions: by assessing performance in a Bayesian statistical framework, we have strengthened the quantitative evidence for the Bayesian model for pronouns. By testing German personal and demonstrative pronouns, we have extended cross-linguistic support for the Bayesian model and also applied it to a type of pronoun with a more rigid interpretation bias, showing the model’s flexibility, while at the same time providing new insights into the comprehension of the German demonstrative *dieser*. The study also provides evidence in favor of the strong form of the Bayesian model, with verb type affecting the prior but not the production likelihoods of personal and demonstrative pronouns separately. However, given that overall, model performance was negatively affected by the removal of verb type information from the production likelihoods, there is room for speculation that the dissociation of factors in the strong form of the Bayesian model could be moderated. Finally, the study was set up to provide clearer evidence about the role of agentivity versus subjecthood on the pronoun production likelihoods, but we are unable to draw strong conclusions here, and leave this question for future research.

## Data Availability Statement

The datasets presented in this study can be found in online repositories. The names of the repository/repositories and accession number(s) can be found below: Center for Open Science (OSF): osf.io/j5wtg.

## Ethics Statement

The studies involving human participants were reviewed and approved by Ethikkommission der Deutschen Gesellschaft für Sprachwissenschaft. The participants provided their written informed consent to participate in this study.

## Author Contributions

CP contributed to data curation, formal analysis, investigation, methodology, project administration, supervision, validation, writing – original draft, and writing – review and editing. PS contributed to conceptualization, formal analysis, funding acquisition, project administration, supervision, and writing – review and editing. BN contributed to formal analysis, methodology, visualization, and writing – review and editing. JH contributed to data curation, investigation, methodology, resources, validation, and writing – original draft. AK contributed to conceptualization, methodology, and writing – review and editing. All authors contributed to the article and approved the submitted version.

## Conflict of Interest

The authors declare that the research was conducted in the absence of any commercial or financial relationships that could be construed as a potential conflict of interest.

## Publisher’s Note

All claims expressed in this article are solely those of the authors and do not necessarily represent those of their affiliated organizations, or those of the publisher, the editors and the reviewers. Any product that may be evaluated in this article, or claim that may be made by its manufacturer, is not guaranteed or endorsed by the publisher.

## Footnotes

1 This is similar to what happens when data are averaged for a *t*-test in comparison to using the “raw” data in a linear mixed model.

2 In order to refer to propositional content (for example an aforementioned sentence) speakers of German use the neuter form of pronouns (*das*, *dies*), similar to English *this* and *that* (see Çokal et al., 2018).

3 This methodology differs from previous story completion experiments testing the Bayesian model, because different sets of participants took part in the pronoun-prompt and free-prompt tasks.

4 Predictions and observations were on a per-condition/pronoun basis rather than an item and/or participant basis, so a total of 16 observation pairs were used for the correlation.

5 We follow Dowty’s (1991) use of proto-roles.

6 It should be noted, however, that Rohde and Kehler (2014) found no influence of thematic role on production likelihoods when testing active versus passive structures.

7 Wiemer (1996, p. 85) indicates that pejorative use is a potential additional function of the *der*-type pronoun. Bethke (1990, p. 72) points out that the *der*-type pronoun is not only used in negatively connoted situations and claims that the pejorative use results from other linguistic and contextual factors. Corpus research reports very few cases of *der* with (mild) pejorative connotations: e.g., in the course books of Eurolingua 2 out of 936 instances of the *der*-type pronoun are pejorative (Ahrenholz, 2007, p. 338). The pejorative connotation might further be intertwined with contrast. Sometimes *dieser* is also associated with pejorative use.

8
http://osf.io/j5wtg

9 This is because the number of dative verbs in German is restricted: in previous experiments (Fuchs, 2021) only four dative verbs (*gefallen* “to please,” *missfallen* “to displease,” *auffallen* “to notice,” and *imponieren* “to impress”) were interpreted correctly and hence used in the present experiment.

10 We used: R (Version 4.0.3; R Core Team, 2020) and the R packages *bayesplot* (Version 1.7.2; Gabry et al., 2019), *cmdstanr* (Version 0.3.0; Gabry and Češnovar, 2020), *dplyr* (Version 1.0.2; Wickham et al., 2020), *ggplot2* (Version 3.3.3; Wickham, 2016), *kableExtra* (Version 1.3.1; Zhu, 2020), *loo* (Version 2.3.1.9000; Vehtari et al., 2017; Yao et al., 2017), *matrixStats* (Version 0.57.0; Bengtsson, 2020), *posterior* (Version 0.1.3; Vehtari et al., 2020), *purr* (Version 0.3.4; Henry and Wickham, 2020), and *tidyr* (Version 1.1.2; Wickham, 2020).

11 The pointwise log predictive density is proportional to the MSE if the model is normal with constant variance, but it is also appropriate for models that are not normally distributed (Gelman et al., 2013, ch. 7).

12 The tabulated data can be found in Supplementary Material.

13 Full model specification and outputs can be found in Supplementary Material.

14 Indeed, it is not surprising that the Mirror model is a lot closer to the Bayesian model and the actual data for the demonstrative pronouns than the Expectancy model. The Expectancy model resolves the pronoun to the referent that is most expected; one of the functions of demonstrative pronouns is to highlight a less expected referent. Hence, no-one would claim that the Expectancy model as it is implemented here can be applied to demonstratives. Nevertheless, looking only at personal pronouns, the Bayesian model still outperforms the Expectancy model.

15 But see Dowty (1991, p. 579) for competition between agentive features in SE contexts where the stimulus entails the proto-agent property causation and the experiencer entails sentience.

16 Analysis and outcome for the dative items can be found on OSF (osf.io/j5wtg). The overall pattern for the dative items was similar to Experiment 1.

17 The tabulated data can be found in Supplementary Material.

18 Furthermore, we tested whether participants had the same interpretations of the SE *dieser–*NP1 completions as our annotators; participants agreed with our annotations (by choosing NP1) on average 71% of the time. The probability of NP1 choice, calculated per item, did not have a significant influence on the ratings of the SE *dieser–*NP1 completions (*z* = −0.26).

19 As a reminder, in ES constructions subjects and experiencers (as the highest thematic role) are aligned, while in SE constructions NP1 outranks NP2 with respect to subjecthood but NP2 outranks NP1 with respect to agentivity. Thus if agentivity and subjecthood both have a strong influence on likelihoods, the NP1–NP2 difference should be stronger in the ES constructions than in the SE constructions.
