# Supplementary material for: A Bayesian Approach to German Personal and Demonstrative Pronouns
Source: Front Psychol. 2022 Mar 3;12:672927. doi: 10.3389/fpsyg.2021.672927 (PMC8927811; doi:10.3389/fpsyg.2021.672927)
Supplement: Supplementary file 1 [file Data_Sheet_1.docx]

### Data overview, Experiment 1

**Table 1. Distribution of reference in the pronoun prompt conditions, Experiment 1.**

| **Reference** | **Number of cases** | **Percentage of total** |
| --- | --- | --- |
| NP1 | 522 | 22.7 |
| NP2 | 1189 | 51.6 |
| Ambiguous | 513 | 22.3 |
| Ungrammatical | 9 | 0.4 |
| Data missing | 71 | 3.1 |
| **TOTAL** | **2304** |  |

**Table 2. Distribution of reference in the free prompt condition, Experiment 1.**

| **Reference** | **Number of cases** | **Percentage of total** |
| --- | --- | --- |
| NP1 | 249 | 21.6 |
| NP2 | 430 | 37.3 |
| Both | 145 | 12.6 |
| Neither | 111 | 9.6 |
| Ambiguous | 129 | 11.2 |
| Ungrammatical | 38 | 3.3 |
| Data missing | 50 | 4.3 |
| **TOTAL** | **1152** |  |

**Table 3. Distribution of response categories of first referential expression in free prompt condition, Experiment 1.**

| **Response Category** | **Number of cases** | **Percentage of total** |
| --- | --- | --- |
| Personal pronoun ER subject | 537 | 46.6 |
| Personal pronoun non-subject | 23 | 2.0 |
| Demonstrative DIESER subject | 160 | 13.9 |
| Demonstrative DIESER non-subject | 4 | 0.3 |
| Demonstrative DER subject | 14 | 1.2 |
| Demonstrative DER non-subject | 1 | 0.1 |
| Plural pronouns – personal  (all grammatical roles) | 101 | 8.8 |
| Plural pronouns – demonstrative  (all grammatical roles) | 3 | 0.2 |
| Pronoun – other  (e.g. indefinite pronoun “beide”) | 43 | 3.7 |
| NP (definite; indefinite), subject | 115 | 10.0 |
| NP (definite; indefinite), non-subject | 5 | 0.4 |
| Plural NP | 2 | 0.2 |
| Impersonal pronouns (es, man) | 27 | 2.3 |
| Complex anaphora (e.g. das, dies referring to whole proposition or event) | 23 | 2.0 |
| No referential expression | 6 | 0.5 |
| Ungrammatical | 38 | 3.3 |
| Missing data | 50 | 4.3 |
| **TOTAL** | **1152** |  |

**Table 4. Next-mention biases (proportion of continuations about NP1) per verb-type and prompt for Experiment 1.**

| **Verb type** | **Prompt** | **Number of cases** | **Raw proportion** |
| --- | --- | --- | --- |
| Accusative | Dieser | 24/402 | 0.06 |
| Accusative | Er | 280/395 | 0.71 |
| Accusative | Free | 121/289 | 0.42 |
| Dative | Dieser | 53/451 | 0.12 |
| Dative | Er | 165/463 | 0.36 |
| Dative | Free | 128/390 | 0.33 |

**Table 6. Probability of using a personal pronoun to refer to NP1 and NP2, per verb type**

| Verb type | Referent | Use of personal pronoun | No use of personal pronoun | Total cases | Personal pronoun bias |
| --- | --- | --- | --- | --- | --- |
| Accusative | NP1 | 107 | 14 | 121 | 0.88 |
| Accusative | NP2 | 47 | 121 | 168 | 0.28 |
| Dative | NP1 | 121 | 7 | 128 | 0.95 |
| Dative | NP2 | 183 | 79 | 262 | 0.70 |

**Table 7. Probability of using a demonstrative pronoun to refer to NP1 and NP2, per verb type**

| Verb type | Referent | Use of demonstrative pronoun | No use of demonstrative pronoun | Total cases | Demonstrative pronoun bias |
| --- | --- | --- | --- | --- | --- |
| Accusative | NP1 | 3 | 118 | 121 | 0.02 |
| Accusative | NP2 | 76 | 92 | 168 | 0.45 |
| Dative | NP1 | 2 | 126 | 128 | 0.02 |
| Dative | NP2 | 71 | 191 | 262 | 0.27 |

Figure S1. By-participant predictions and observed responses, Experiment 1. The observed proportion of responses (from held out data) are depicted with black crosses; distribution of simulated proportions based on the model predictions are depicted with box plots.

Figure S2. By-item predictions and observed responses, Experiment 1. The observed proportion of responses (from held out data) are depicted with black crosses; distribution of simulated proportions based on the model predictions are depicted with box plots.

Brm model outputs for testing the influence of verb type and agentivity on production likelihoods for the demonstrative pronoun, Experiment 1.

brm(dem_pron ~ verbtype.sum * referent.sum

+ (1 | participant)

+ (1 | Item),

data=zero_trimmed,

family = bernoulli(link = logit),

prior =

c(prior(normal(0, 2), class = Intercept),

prior(normal(0, 1), class = b),

prior(normal(0, 1), class = sd)),

control = list(adapt_delta = 0.9)

)

|  | Estimate | Estimate Error | 95% Credible Interval lower bound | 95% Credible Interval upper bound |
| --- | --- | --- | --- | --- |
| Intercept | -2.54 | 0.29 | -3.15 | -2.00 |
| Verb Type | 0.33 | 0.23 | -0.11 | 0.80 |
| Referent | -1.74 | 0.23 | -2.24 | -1.31 |
| Verb Type x Referent | -0.12 | 0.23 | -0.56 | 0.34 |

Brm model outputs for testing the influence of verb type and agentivity on production likelihoods for the personal pronoun, Experiment 1.

Model spec:

brm(pers_pron ~ verbtype.sum * referent.sum

+ (1 | participant)

+ (1 | Item),

data=zero_trimmed,

family = bernoulli(link = logit),

prior =

c(prior(normal(0, 2), class = Intercept),

prior(normal(0, 1), class = b),

prior(normal(0, 1), class = sd)),

control = list(adapt_delta = 0.9)

)

|  | Estimate | Estimate Error | 95% Credible Interval lower bound | 95% Credible Interval upper bound |
| --- | --- | --- | --- | --- |
| Intercept | 1.42 | 0.22 | 1.01 | 1.86 |
| Verb Type | -0.75 | 0.15 | -1.04 | -0.45 |
| Referent | 1.46 | 0.15 | 1.18 | 1.78 |
| Verb Type x Referent | 0.24 | 0.14 | -0.03 | 0.52 |

## Data Overview Experiment 2

**Table 1. Distribution of reference in the pronoun prompt conditions, Experiment 2.**

| **Reference** | **Number of cases** | **Percentage of total** |
| --- | --- | --- |
| NP1 | 395 | 41.1 |
| NP2 | 474 | 49.4 |
| Ambiguous | 81 | 8.4 |
| Ungrammatical | 9 | 0.9 |
| Data missing | 1 | 0.1 |
| **TOTAL** | **960** |  |

**Table 2. Distribution of reference in the free prompt condition, Experiment 2.**

| **Reference** | **Number of cases** | **Percentage of total** |
| --- | --- | --- |
| NP1 | 189 | 39.4 |
| NP2 | 163 | 34.0 |
| Both | 11 | 2.3 |
| Neither | 65 | 13.5 |
| Ambiguous | 13 | 2.7 |
| Ungrammatical | 37 | 7.7 |
| Data missing | 2 | 0.4 |
| **TOTAL** | **480** |  |

**Table 3. Distribution of response categories of first referential expression in free prompt condition, Experiment 2.**

| **Response Category** | **Number of cases** | **Percentage of total** |
| --- | --- | --- |
| Personal pronoun ER subject | 238 | 49.6 |
| Personal pronoun non-subject | 30 | 6.3 |
| Demonstrative DIESER subject | 70 | 14.6 |
| Demonstrative DIESER non-subject | 1 | 0.2 |
| Demonstrative DER subject | 4 | 0.8 |
| Demonstrative JENER subject | 1 | 0.2 |
| Plural pronouns – personal  (all grammatical roles) | 5 | 1.0 |
| Pronoun – other  (e.g. indefinite pronoun “beide”) | 9 | 1.9 |
| NP (definite; indefinite), subject | 48 | 10 |
| NP (definite; indefinite), non-subject | 7 | 1.5 |
| Plural NP | 2 | 0.4 |
| Impersonal pronouns (es, man) | 8 | 1.7 |
| Complex anaphora (e.g. das, dies referring to whole proposition or event) | 11 | 2.3 |
| No referential expression | 7 | 1.5 |
| Ungrammatical | 37 | 7.7 |
| Missing data | 2 | 0.4 |
| **TOTAL** | **480** |  |

**Table 4. Next-mention bias for NP1 per verb-type and prompt for Experiment 2**

| **Verb type** | **Prompt** | **Number of cases** | **Raw proportion** |
| --- | --- | --- | --- |
| E–S | Dieser | 0/215 | 0 |
| E–S | Er | 120/210 | 0.57 |
| E–S | Free | 67/175 | 0.38 |
| S–E | Dieser | 68/215 | 0.32 |
| S–E | Er | 207/229 | 0.90 |
| S–E | Free | 122/177 | 0.69 |

**Table 6. Probability of using a personal pronoun to refer to NP1 and NP2, per verb type for Experiment 2**

| Verb type | Referent | Use of personal pronoun | No use of personal pronoun | Total cases | Personal pronoun bias |
| --- | --- | --- | --- | --- | --- |
| E–S | NP1 | 67 | 0 | 67 | 1 |
| E–S | NP2 | 54 | 54 | 108 | .50 |
| S–E | NP1 | 116 | 6 | 122 | .95 |
| S–E | NP2 | 19 | 36 | 55 | .35 |

**Table 7. Probability of using a demonstrative pronoun to refer to NP1 and NP2, per verb type for Experiment 2**

| Verb type | Referent | Use of demonstrative pronoun | No use of demonstrative pronoun | Total cases | Demonstrative pronoun bias |
| --- | --- | --- | --- | --- | --- |
| E–S | NP1 | 0 | 67 | 67 | 0 |
| E–S | NP2 | 41 | 67 | 108 | .38 |
| S–E | NP1 | 3 | 119 | 122 | .02 |
| S–E | NP2 | 29 | 26 | 55 | .53 |

Figure S3. By-participant predictions and observed responses, Experiment 2. The observed proportion of responses (from held out data) are depicted with black crosses; distribution of simulated proportions based on the model predictions are depicted with box plots.

Figure S4. By-item predictions and observed responses, Experiment 2. The observed proportion of responses (from held out data) are depicted with black crosses; distribution of simulated proportions based on the model predictions are depicted with box plots.

Brm model outputs for testing the influence of verb type, and the relative contribution of agentivity and subjecthood, on production likelihoods for the demonstrative pronoun, Experiment 2.

brm(dem_pron ~ verbtype.sum * referent.sum

+ (1 | subj)

+ (1 | item_nr),

data=zero_trimmed,

family = bernoulli(link = logit),

prior =

c(prior(normal(0, 2), class = Intercept),

prior(normal(0, 1), class = b),

prior(normal(0, 1), class = sd)),

control = list(adapt_delta = 0.9)

)

|  | Estimate | Estimate Error | 95% Credible Interval lower bound | 95% Credible Interval upper bound |
| --- | --- | --- | --- | --- |
| Intercept | -2.77 | 0.48 | -3.83 | -1.89 |
| Verb Type | -0.49 | 0.35 | -1.22 | 0.17 |
| Referent | -2.37 | 0.36 | -3.13 | -1.71 |
| Verb Type x Referent | -0.18 | 0.34 | -0.89 | 0.45 |

Brm model outputs for testing the influence of verb type, and the relative contribution of agentivity and subjecthood, on production likelihoods for the personal pronoun, Experiment 2.

brm(pers_pron ~ verbtype.sum * referent.sum

+ (1 | subj)

+ (1 | item_nr),

data=zero_trimmed,

family = bernoulli(link = logit),

prior =

c(prior(normal(0, 2), class = Intercept),

prior(normal(0, 1), class = b),

prior(normal(0, 1), class = sd)),

control = list(adapt_delta = 0.9)

)

|  | Estimate | Estimate Error | 95% Credible Interval lower bound | 95% Credible Interval upper bound |
| --- | --- | --- | --- | --- |
| Intercept | 1.99 | 0.39 | 1.29 | 2.81 |
| Verb Type | 0.62 | 0.32 | 0.06 | 1.27 |
| Referent | 2.35 | 0.33 | 1.77 | 3.05 |
| Verb Type x Referent | 0.35 | 0.31 | -0.23 | 1.00 |

**Follow-up rating experiment**

The follow-up rating experiment (see Experiment 2 discussion) was a combined rating task and antecedent choice task. The purpose of this experiment was to check our intuition that the higher-than-expected number of references to NP1 with the demonstrative in SE conditions of Experiment 2 were due to an artefact of the factorial design; in the SE contexts with a *dieser* prompt, the participant is faced with two options for interpretation of *dieser*, both of which are sub-optimal. Referring to NP1 with *dieser* is not felicitous because this goes against the preferences for the demonstrative, which is normally resolved to a less prominent antecedent. Referring to NP2, however, works against the strong bias coming from the SE verb to talk about the stimulus (i.e. NP1), rather than the experiencer (NP2). Given this conflict, we surmised that participants often made the choice to talk about the stimulus and thus refer to NP1 even though this choice is not ideal.^[[1]](#footnote-1)^ If our suspicion is correct, the completions in which *dieser* refers to NP1 in the SE condition should be less felicitous than completions in which *dieser* refers to NP2 and completions in which *er* refers to NP1. Experiment 3 was set up to test this hypothesis. In this experiment, a new set of participants rated a subset of the SE completions that were provided by participants in the pronoun prompt conditions in Experiment 2. We additionally tested whether these new participants agreed with our annotations in those cases where *dieser* is annotated as referring to NP1 in SE contexts, by asking them to select an antecedent. This is also a crucial check for interpreting the ratings, because if participants thought that *dieser* was referring to NP2 instead of NP1 in a particular completion, they would (presumably) rate that completion as acceptable.

**Participants**

45 participants were recruited via the online platform Prolific.ac to take part in the follow-up experiment. All participants gave their consent and received a small fee for participation. None had taken part in Experiment 2. All participants indicated that they were native German speakers, and no participant reported a language disorder. Three participants were excluded because they were bilingual. Three further participants were excluded because they did not perform the task properly.^[[2]](#footnote-2)^ Data from the remaining 39 participants (age range 18-68, mean age 33 years; 24 male, 15 female) were used in the analysis.

**Materials**

There were 68 SE dieser–NP1 completions in Experiment 2. For the rating task, these 68 completions were divided over 4 lists (in order not to tire participants by presenting too many completions), each containing 17 completions. The remainder of the completions were the same across all lists to give a stable comparison point. Each participant saw one list. Each list was composed as follows:

- 17 SE *dieser*–NP1 completions from Experiment 2: this was a subset of the 68 SE *dieser*–NP1 completions
- 17 SE *dieser*–NP2 completions: these were chosen at random from all available SE *dieser*–NP2 completions from Experiment 2
- 17 SE *er*–NP1 completions: these were chosen at random from all available SE *er*–NP1 completions from Experiment 2
- 24 fillers (of which 12 are nonsense fillers to probe use of the extremes of the scale)

For the forced referent choice task, participants saw the same 17 SE *dieser*–NP1 items that they saw in the rating task, with 17 fillers (8 *dieser*–NP2 and 9 *er*–NP1 items).

**Procedure**

Based on a short description of the task, participants could choose to take part in the study via the Prolific.ac application. Participants were then directed to the Qualtrics platform and gave their consent and answered a short series of biographical questions before starting the experimental task. For task 1 (rating), participants were instructed to read each text and to rate how the text sounds for them on a response scale. The scale had five points, for which only text labels (no numerical values) were displayed. The labels were “sehr schlecht” (*very bad*), “eher schlecht” (*quite bad*), “neutral” (*neutral*), “eher gut” (*quite good)* and “sehr gut” (*very good*). The scale was always presented in the same direction, with “sehr schlecht” on the left. For task 2 (referent choice), participants were instructed to read each text and decide on the referent for the pronoun; this instruction was clarified by displaying an example, and by following each text with a question about the pronoun. The question was always a wh-question version of the pronoun sentence. For example, if the text was “The teacher praised the pupil. He was very hardworking.”, the question following the text would be “Who was very hardworking?”. The two referent options were displayed below the question.

**Data analysis**

An analysis plan for the data was pre-registered on aspredicted.org. The registration can be found in Supplementary Materials. Rating data (task 1) were analysed using a cumulative-link model using the package *ordinal* (Christensen, 2019) in RStudio (RStudio Team 2019) using R version 3.6.1 (R Core Team 2019). Completion type (*dieser*–NP1; *dieser*–NP2; *er*–NP1) was a fixed effect, and a random effect for participant was included. Referent choice data was summarised as a proportion of NP1 referent choices per item, and this was used as a fixed effect in a cumulative link model that looked only at the dieser-NP1 items.

**Results**

The number of responses in each rating category is given in the table below.

Number of responses in each rating category per completion type, follow-up experiment.

| Response | *dieser*–NP1 | *dieser*–NP2 | *er*–NP1 |
| --- | --- | --- | --- |
| Very bad | 43 | 18 | 15 |
| Quite bad | 229 | 55 | 59 |
| Neutral | 104 | 103 | 97 |
| Quite good | 141 | 242 | 225 |
| Very good | 146 | 245 | 267 |

The cumulative link model showed that both *dieser*–NP2 completions and the *er*–NP1 completions were significantly more likely to elicit better ratings than *dieser*–NP1 completions (*z* = 11.52 for *dieser*–NP2 and 12.28 for *er*–NP1).

In the referent choice task, participants agreed with our annotations (by choosing NP1) on average 71% of the time. The probability of NP1 choice was calculated on a per item basis, and this probability was used as a predictor in a cumulative link model of the ratings of the *dieser*–NP1 completions from task 1. The probability of NP1 choice did not have a significant influence on the ratings (*z*=-0.26).

1. This type of conflict (where both reference options are less than felicitous) is not present in the other conditions. The flexibility of *er* means that such a conflict does not arise in the *er* prompts, and in the ES contexts with *dieser* prompts the verb bias and the pronoun bias work in the same direction, in favour of NP2. [↑](#footnote-ref-1)
2. This was determined by comparing individual participants’ ratings on the nonsense fillers to their ratings on the rest of the filler items (‘normal’ fillers); those who rated the nonsense fillers the same as or better than the normal fillers were excluded. [↑](#footnote-ref-2)
